# Genome-wide DNA methylation analysis reveals molecular subtypes of pancreatic cancer

**DOI:** 10.18632/oncotarget.15993

**Published:** 2017-03-07

**Authors:** Nitish Kumar Mishra, Chittibabu Guda

**Affiliations:** ^1^ Department of Genetics, Cell Biology and Anatomy, University of Nebraska Medical Center, Omaha, NE, 68198, USA; ^2^ Bioinformatics and Systems Biology Core, University of Nebraska Medical Center, Omaha, NE, 68198, USA; ^3^ Department of Biochemistry and Molecular Biology, University of Nebraska Medical Center, Omaha, NE, 68198, USA; ^4^ Fred and Pamela Buffet Cancer Center, University of Nebraska Medical Center, Omaha, NE, 68198, USA

**Keywords:** TCGA, pancreatic cancer, differential methylation, integrative analysis, molecular subtypes

## Abstract

Pancreatic cancer (PC) is the fourth leading cause of cancer deaths in the United States with a five-year patient survival rate of only 6%. Early detection and treatment of this disease is hampered due to lack of reliable diagnostic and prognostic markers. Recent studies have shown that dynamic changes in the global DNA methylation and gene expression patterns play key roles in the PC development; hence, provide valuable insights for better understanding the initiation and progression of PC. In the current study, we used DNA methylation, gene expression, copy number, mutational and clinical data from pancreatic patients. We independently investigated the DNA methylation and differential gene expression profiles between normal and tumor samples and correlated methylation levels with gene expression patterns. We observed a total of ~23-thousand differentially methylated CpG sites (Δβ≥0.1) between normal and tumor samples, where majority of the CpG sites are hypermethylated in PC, and this phenomenon is more prominent in the 5′UTRs and promoter regions compared to the gene bodies. Differential methylation is observed in genes associated with the homeobox domain, cell division and differentiation, cytoskeleton, epigenetic regulation and development, pancreatic development and pancreatic signaling and pancreatic cancer core signaling pathways. Correlation analysis suggests that methylation in the promoter region and 5′UTR has mostly negative correlations with gene expression while gene body and 3′UTR associated methylation has positive correlations. Regulatory element analysis suggests that HOX cluster and histone core proteins are upstream regulators of hypomethylation, while SMAD4, STAT4, STAT5B and zinc finger proteins (ZNF) are upstream regulators of hypermethylation. Non-negative matrix factorization (NMF) clustering of differentially methylated sites generated three clusters in PCs suggesting the existence of distinct molecular subtypes. Cluster 1 and cluster 2 showed samples enriched with clinical phenotypes like neoplasm histological grade and pathologic T-stage T3, respectively, while cluster 3 showed the enrichment of samples with neoplasm histological grade G1. To the best of our knowledge, this is the first genome-scale methylome analysis of PC data from TCGA. Our clustering analysis provides a strong basis for future work on the molecular subtyping of epigenetic regulation in pancreatic cancer.

## INTRODUCTION

Pancreatic cancer (PC) is the fourth leading cause of cancer deaths today in the United States [1] and it is poised to become the second leading cause within a decade [2] due to the aggressiveness of the disease and the lack of reliable early diagnostic markers. Survival prospects of many other cancers have improved over the past decade, but only a marginal change was observed in pancreatic cancer survival. Pancreatic cancer patients have a median survival of 6 months and a 5-year survival rate of only 6% despite 50 years of research and therapeutic developments [3]. The lack of specific symptoms at early stages of tumor initiation, high biological aggressiveness of the tumor and resistance to cytotoxic drugs, all contribute to the high mortality rate of PC. In nearly 95% of PC patients there is neither an associated family history of PC nor of diseases known to be associated with an increased risk of PC [4]. This points out to the fact that the etiology of PC is more sporadic than genetic, suggesting that epigenetic alterations may play a key role in the transcriptional regulation of genes in pancreatic cancer.

Epigenetic alteration of gene expression can be achieved by DNA methylation, which is known to be deregulated in pancreatic cancer resulting in altered gene expression [5, 6], genome structure reorganization, tumor grade, tumor stage and survival time of patients [3]. The Cancer Genome Atlas (TCGA) [7, 8] and International Cancer Genome Consortium (ICGC) [9] have generated methylome data for thousands of tumor samples spanning across ~25 cancer types including pancreatic cancer. The large number of available TCGA tumor datasets provides the opportunity to study the global methylation and gene expression patterns of PCs with increased statistical power, which would not have been possible otherwise. Several DNA methylation pattern analyses were previously reported for many cancer types using TCGA data [10–13]; but to our knowledge no such report exists for genome scale methylation pattern analysis of PCs using TCGA datasets.

Earlier studies on PC methylation analysis have used limited patient samples or CpG sites. Sato *et. al*. [14] analyzed genome-scale DNA methylation patterns in PC, but the limitation of this study is that they used methylation-site-specific polymerase chain reaction for only eight genes. Later, Tan *et. al*. [15] studied the methylation patterns of PC using data from thirty xenografts, seven adjacent normal tissues and fourteen human PC cell lines. This study used the Illumina GoldenGate Methylation Cancer Panel, which contains 1,536 CpG sites distributed across only 807 genes with low genome coverage. Recently, Thompson *et. al*. [3] used Illumina Hiseq 2000 methylation data from 16 samples (11 tumor, 2 normal and 3 chronic pancreatitis sample) to analyze the role of DNA methylation in the survival of PC patients. However, this is a focused study on correlating DNA methylation with patient's survival using a small dataset. Similarly, Nones *et. al*. [16] used HumanMethylation450 BeadChip data from ICGC to analyze genome-wide methylation patterns in PC. This study used simple Spearman's correlation of differentially methylated CpGs to integrate DNA methylation and gene expression data at the gene-level, whereas, we are using TCGA data with special emphasis on correlating methylations patterns of genes (100KB up and downstream of transcription start site (TSS)) with gene expressions, which is biologically more relevant. We are also looking at the methylation patterns at the distal enhancer region, probable upstream regulator and epigenetic-driven gene in TCGA PC. Most recently Kim *et. al*. [17] published on the pan cancer DNA methylation analysis of TCGA data, but this study uses only twenty three imprinted genes.

Epigenetic changes such as DNA methylation effect gene regulation in normal development. In normal cells, DNA methylation assures proper regulation of gene expression and stable gene silencing leading to physiological homeostasis. Epigenetic signatures are acquired by cells during cancer tumorogenesis [18], which in turn allows them to overcome physiological homeostasis. These properties confer cells with continuous proliferation potential, self-sufficiency in growth and apoptotic signals and ability to evade the immune system [19]. Transcriptional silencing or enhancement of critical growth regulators through promoter hypermethylation or hypomethylation, respectively, also plays a major role in cancer. Hedgehog and notch signaling pathways are very important for proper development of pancreas. Reactivation of both of these pathways is common in pancreatic cancer [20, 21]. Recent work by Thompson *et. al*. [3] also suggests that differential methylation of pancreas development related genes including homeobox-containing genes are important for PC development and survival of PC patients. Yang *et. al*. [13] used TCGA data for ten major cancer types and observed that methylation and expression patterns of epigenetic enzymes can play a major role in many cancers, but this study did not include data from PC patients.

Given the increasing evidence on the role of epigenetics in cancer, we hypothesize that epigenetic landscape of PC tumors would be distinct compared to those of the normal tissues and that these epigenetic alterations can be correlated with the gene expression patterns in PCs. It has been shown that DNA methylation and gene expression of homeobox-containing genes, pancreas development genes and epigenetic regulatory genes play a vital role in PC [22]. In the current study, we carried out detailed analysis of the global differential methylation patterns in individual chromosomes, homeobox containing gene family and genes involved in epigenetic regulation, and clustered the patient population based on these patterns. We correlated the clustered groups with somatic mutation loads and copy number variations observed in important oncogenes and tumor suppressor genes. We also made detailed correlations between methylation patterns and gene expression levels using methylation data from different sub-regions of genes. Pathway and gene ontology (GO) enrichment analysis of differentially methylated and differentially expressed genes (DEG) enabled us to understand how changes in methylation affect the biological pathways involved in the progression of PC. We also examined the correspondence between pancreatic cancer-specific hypermethylated and hypomethylated distal enhancer probes and the known transcription factor binding motifs to obtain details about which site-specific transcription factors may be involved in the development and progression of PC.

## RESULTS

For pancreatic cancer data analysis we downloaded DNA methylation, gene expression, copy number, somatic mutation and clinical data from TCGA. In the current work, first we investigated the global patterns of DNA methylation and the affected genes and pathways in PC by using tumor and normal samples. We also investigated the methylation patterns of homeobox-containing genes, pancreas development genes and epigenetic regulatory enzymes to understand their role in PC development. Further, we looked at the differential gene expression patterns, enrichment analysis of differentially expressed genes and correlated the DNA methylation and gene expression in PC. Apart from these analyses, we also looked DNA methylation in distal enhancer region and possible molecular subtypes in TCGA PC by using DNA methylation data. R/Bioconductor tools and R function were used for all these analysis in R version 3.3.1.

### Batch effect in TCGA data

High throughput data generation is prone to have batch effect due to variations in the equipment and/or reagent kits used at different locations or the skill level of the handling personnel, etc. As TCGA samples are processed in batches rather than a single run at different sites of the consortium, the data can be vulnerable to batch effects. Hence, we carried out Principal Component Analysis (PCA) by using Mbatch [23] to identify potential batch effects among the data samples and found no batch effect either in the DNA methylation (Supplementary Figure 1) or in the gene expression data (Supplementary Figure 2).

### Global DNA methylation analysis

**Figure 1:**
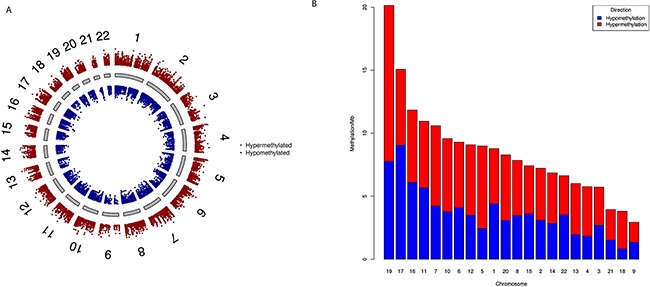
Genome-wide differential DNA methylation patterns in pancreatic cancer **(A)** Difference in DNA methylation in all CpG sites passing false discovery rate (FDR) with Δβ ≥ 0.1. Δβ is weighted by *T*-statistics such that the distance from central core (grey) indicates increasing level of statistical significance. Chromosomes are shown in clockwise from 1 to 22; we did not use sex chromosomes (X or Y) in our analysis. **(B)** Chromosome wise DNA methylation frequency distribution in pancreatic cancer. For each chromosome we calculated hypermethylation and hypomethylation frequency per megabase pairs. We sorted chromosomes on the basis of DNA methylation/Mb. Chromosome 4, 5, 13 and 18 have two-fold higher hypermethylation frequencies.

To study the global methylation patterns in PC, we carried out differential methylation analysis of level-3 PC data from TCGA. We observed a total of 23,688 CpG sites that are differentially methylated (Δβ≥0.1) between tumor and normal samples; out of these 13,501 are hypermethylated and 10,187 are hypomethylated (Supplementary Data 1). At higher thresholds, the number of differentially methylated CpG sites (henceforth referred to as dm-CpGs) fall sharply to 11,480 (Δβ≥0.2) or 2,751 (Δβ≥0.3). Figure 1A shows all the dm-CpGs on each chromosome at Δβ≥0.1. We observed that chromosomes 1 and 2 have maximum number of dmCpGs, while chromosomes 21 and 18 have the least. This is expected given the differences in the relative sizes of these chromosomes. Further, we also calculated the methylation frequency per Mb for each chromosome to determine the net changes in the dm-CpGs (Figure 1B), Chromosomes 19, 17, 16, 11 and 7 in that order show high frequency of dm-CpGs (>10 dm-CpGs/Mb). Chromosome 19 has the highest at 20.14 dm-CpGs/Mb while chromosome 9 has the lowest with only 2.39 dm-CpGs/Mb (Supplementary Data 2). Except chromosomes 1, 11, 16 and 22, all chromosomes have more hypermethylated CpG sites than hypomethylated CpGs. Of note, chromosomes 4, 5, 13 and 18 showed more than two-fold hypermethylation frequency (Supplementary Data 2). We also observed that CpG islands, shores and shelfs regions are predominantly hypermethylated, while open sea regions are hypomethylated (Supplementary Data 3).

At the gene level, 7,405 genes have dm-CpGs at Δβ≥0.1 with a total of 1,351 genes have at least five or more dm-CpGs (Supplementary Data 1). Table 1 gives the details on the number of dm-CpG sites in different gene subregions and the number of genes corresponding to these probes. Table 2 has the list of top twenty hypermethylated and hypomethylated CpG site IDs with corresponding gene and chromosome associations. Results indicate that a number of HOX, zinc finger and other transcription factors are significantly differentially methylated in PCs.

**Table 1:** Total number of differentially methylated CpG sites in different sub-regions

CpG subregions	Δβ ≥ 0.1	Δ β ≥ 0.2	Δβ ≥ 0.3
3′UTR	797 (623)	298 (236)	45 (40)
5′UTR	3059 (1563)	1523 (859)	418 (301)
1^st^ Exon	2168 (1086)	1223 (674)	407 (274)
Gene Body	8593 (3663)	3731 (1962)	757 (530)
TSS200	2292 (1054)	1249 (592)	373 (224)
TSS1500	3125 (1691)	1318 (763)	294 (209)
TSS1.5Kb	9465 (3167)	4669 (1719)	1243 (638)

The numbers in parenthesis represent the total number of HGNC genes covered by these CpG sites.

UTR- Untranslated Region; TSS- Transcription Start Site, TSS1.5Kb- 1.5 Kb up and downstream from TSS

**Table 2:** Top twenty hypermethylated and hypomethylated CpG sites in known genes

Illumina probe id	Δβ value	Chromosome	Gene symbol	Fold change
cg22674699	0.60	chr2	HOXD9	4.713211
cg03692651	0.59	chr19	ZNF729	3.736158
cg03306374	0.57	chr16	PRKCB	6.524701
cg22784954	0.56	chr5	ADAMTS16	4.539619
cg19717586	0.56	chr11	NTM	5.896104
cg16729415	0.55	chr15	GJD2	5.436232
cg15811515	0.55	chr16	YBX3P1	4.782864
cg24221648	0.54	chr13	RNF219-AS1	5.424716
cg17495912	0.54	chr13	CCNA1	4.81373
cg06304097	0.54	chr10	TCERG1L	5.730066
cg26296488	0.53	chr4	DRD5	5.07241
cg22620090	0.53	chr6	LIN28B	3.737363
cg15506157	0.53	chr7	KLRG2	2.38998
cg07915921	0.53	chr12	HOXC13-AS	5.657705
cg22797031	0.52	chr1	PRRX1	3.527956
cg17774559	0.52	chr5	IRX4	4.203264
cg14473102	0.52	chr2	HOXD8	4.714848
cg20302133	0.51	chr1	KCNA3	6.175219
cg17985646	0.50	chr7	TBX20	5.129886
cg25397945	0.49	chr19	ZNF382	5.946388
cg07805542	−0.44	chr1	PIK3CD	0.466222
cg04214938	−0.44	chr2	EN1	0.550001
cg01077100	−0.44	chr10	BTBD16	0.505858
cg13446584	−0.45	chr7	GTF2IRD1	0.548374
cg10728351	−0.45	chr4	ANXA5	0.590933
cg09159452	−0.45	chr7	IQCE	0.540868
cg09077096	−0.45	chr7	CARD11	0.570784
cg21011133	−0.46	chr2	ADCY3	0.434894
cg27411547	−0.47	chr8	SLC45A4	0.587623
cg07388969	−0.47	chr15	SPRED1	0.464429
cg20151476	−0.48	chr7	PSMG3	0.56687
cg07248223	−0.48	chr17	CCR7	0.499523
cg20518446	−0.49	chr11	AHNAK	0.501228
cg20765408	−0.50	chr13	PARP4	0.438326
cg11303839	−0.50	chr7	CCL26	0.505054
cg20928945	−0.53	chr7	ADAP1	0.436731
cg09287864	−0.53	chr7	AHR	0.50094
cg20852851	−0.54	chr2	HDAC4	0.524387
cg05926314	−0.55	chr7	PTPRN2	0.483487
cg23066280	−0.56	chr7	PTPRN2	0.506833

**Figure 2:**
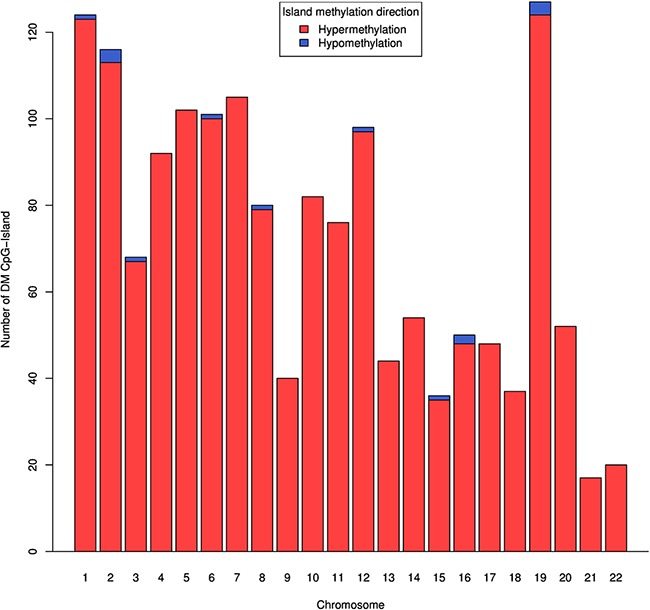
Genome-wide distribution of DNA methylation CpG islands in pancreatic cancer For each known CpG island with more than 3 methylated CpG sites, we calculated median of β value. Difference in DNA methylation in all CpG islands passing FDR with Δβ ≥ 0.1. CpG islands that are hypomethylated are in blue color.

We also analyzed the differential methylation patterns of CpG islands using median β value of CpGs in each CpG island. Only those islands that have at least three CpG sites after preprocessing of data were included in this analysis. We observed a total of 1,570 dm-CpG islands, out of these 1,555 islands are hypermethylated and 15 are hypomethylated (Supplementary Figure 3 and Supplementary Data 4). As shown in Figure 2, the dm-CpG islands are highly enriched in chromosomes 1, 2, 5, 6, 7 and 19, each containing more than hundred dm-CpG islands. We also observed that seven genes encoding epigenetic regulatory enzymes and 29 homeobox-containing genes have one or more dm-CpG islands. A list of top 10 hyper and hypomethylated CpG islands are shown in Table 3.

**Table 3:** Top ten hypermethylated and hypomethylated CpG islands in TCGA pancreatic cancer data

CpG Island	Delta beta	Fold change
chr6:105400877-105401149	047	2.91
chr5:178003623-178004247	0.44	5.16
chr14:57278709-57279116	0.43	5.02
chr7:42267546-42267823	0.40	5.54
chr1:158147433-158147854	0.39	2.15
chr4:5709985-5710495	0.39	3.93
chr2:127413696-127414171	0.39	7.09
chr1:111216244-111217937	0.39	4.87
chr7:19184818-19185033	0.38	4.30
chr1:248020330-248021252	0.38	6.09
chr15:66274583-66274838	−0.18	0.29
chr19:1856725-1857443	−0.18	0.47
chr19:1240154-1240546	−0.18	0.26
chr2:85811340-85811855	−0.20	0.48
chr16:29818681-29819554	−0.20	0.30
chr21:45148454-45149262	−0.24	0.44
chr12:6649677-6649897	−0.25	0.46
chr12:6649677-6649897	−0.27	0.47
chr8:103750881-103751088	−0.31	0.50
chr6:33244677-33245554	−0.33	0.33

### Differential methylation of genes involved in epigenome regulation

DNA methylation and histone modification are epigenetic regulatory mechanisms that affect the overall epigenome and transcriptome landscapes in cancer. These epigenetic regulatory genes are divided into writer, reader and eraser categories based on their mode of action. We analyzed the DNA methylation patterns of each category of epigenetic regulatory genes as well as the differential methylation of core histone proteins and linker proteins in PC.

### Methylation patterns of writer genes

Epigenetic writers catalyze the addition of methyl or acetyl group on DNA or histone proteins. DNA methyl-transferases (DNMT) are writers of epigenome, which can actively methylate cytosine of CpG dinucleotides. DNMT1 methylates CpG sites; we observed hypomethylation of a single CpG site of DNMT1 in PC. In contrast, DNMT3A, a *de-novo* DNA methyltransferase is hypermethylated in PC, but its homologue, DNMT3B has both hyper and hypomethylated CpG sites (Supplementary Data 5). Histone methylation writer can methylate histone proteins, several histone methylation writer genes e.g. euchromatic histone-lysine N-methyltransferases (EHMTs), lysine-specific methyltransferases (KMTs), genes encoding PR domain-contacting proteins (PRDMs) also have dm-CpGs. In addition, we observed differential methylation of other epigenome regulatory writer genes such as EZH2, BRD4, KMT2C/MLL3, KMT2D/MLL2, SMYD2, SMYD3, WHSC1 and WHSC1L1. SET-domain containing histone methylation writer genes such as SETBP1, SETD3, SETD7 and SETMAR were also differentially methylated in PC (Table 4). Differential methylation of histone acetylation writers such as lysine acetyltransferase proteins 2A (KAT2A), KAT6B was also observed in PC. We also observed differential methylation of several other histone acetylation writer genes such as CREBBP, GTF3C1, NCOA1, NCOA2 and NCOA7 (Table 4). Another group of writers, the Arginine methylation writer genes such as protein arginine methyltransferase 6 (PRMT6) and PRMT8 are also differentially methylated in PC (Supplementary Data 5).

**Table 4:** List of differentially methylated epigenetic enzyme, chromatin remodeler and histone proteins in TCGA pancreatic cancer

Mark	Writer	Reader	Eraser/editor
DNA methylation	DNMT1, DNMT3A, DNMT3B	CHD2, CHD7, MBD1, ZBTB38, ZMYM4, ZMYM6,	APOBEC1, IDH2, MGMT, TET3
Histone methylation	EHMT1, EHMT2, EZH2, KMT2C/MML3, KMT2D/MLL2, MECOM, PRDM1, PRDM2, PRDM4, PRDM6, PRDM7, PRDM8, PRDM11, PRDM12, PRDM13, PRDM14, PRDM15, PRDM16, SETBP1, SETD3, SETD7, SETMAR, SMYD2, SMYD3, WHSC1, WHSC1L1	ATXN7, CBX5, CHD2, CHD7, DHX30, DNMT3A, EHMT1, EHMT2, GATAD2A, UHRF1, ZMYM8	KDM2A, KDM2B, KDM3A, KDM3B, KDM4B, KDM6B
Histone acetylation	CREBBP, GTF3C1, KAT2A, KAT6B, NCOA1, NCOA2, NCOA7	ATXN7, BRD1, BRD3, BRD4, DHX30, GATAD2A, ZMYM8	HDAC4, HDAC5, HDAC9, HDAC11, SIRT6, SIRT7
Arginine methylation	PRMT6, PRMT8		
*Chromatin remodeler	ARID1B, CHD2, CHD7, CHD8, DPF3, SMARCA2, SMARCD3, TTF2		
*Histone protein	H1F0, H1FOO, HIST3H2A, HIST1H1E, HIST1H2AG, HIST1H2APS1, HIST1H2BA, HIST3H2BB, HIST1H2BC, HIST2H2BF, HIST1H2BI, HIST1H2BN, HIST1H3B, HIST1H3C, HIST1H3E, HIST1H3F, HIST1H3G, HIST1H3H, HIST1H4F, HIST1H4H		

In this figure we are using green for hypermethylated probes, similarly red for hypomethylated and black color for genes which have both hypermethylated and hypomethylated CpG sites.

*In case of chromatin remodeler and histone proteins there is no reader, writer and eraser

### Methylation patterns of reader genes

Epigenetic readers can recognize epigenetic changes in DNA and histone when they get recruited to that specific site. DNA methylation reader genes such as CHD2, CHD7, MBD1, ZBTB28, ZMYM4 and ZMYM6 are differentially methylated in TCGA PC. Hypermethylation of some histone methylation reader genes such as ATXN7, CHD2, DHX30, euchromatic histone-lysine N-methyltransferase2 (EHMT2), GATAD2A and ZMYM8 was observed, while both hyper and hypomethylated patterns were seed in the other histone methylation reader genes such as CBX5, CHD7, EHMT1 and UHRF1. Bromodomains (BRDs) are epigenetic reader domains that selectively recognize acetylated lysine residues. Our results show that BRD1 and BRD3 have hypomethylated CpGs, while BRD4 is hypermethylated (Table 4).

### Methylation patterns of eraser genes

Epigenetic code eraser genes are very important in cancer; they can erase epigenetic changes and alter gene expression. We observed differential methylation of numerous eraser genes in PCs. DNA methylation eraser genes, APOBEC1 and TET3 are hypomethylated, while other eraser genes, IDH2 and MGMT are hypermethylated. Histone lysine demethylase (KDM) genes such as KDM2A, KDM2B, KDM3B and KDM4B are hypomethylated, while KDM3A and KDM6B are hypermethylated (Table 4). Histone acetylation eraser genes, SIRT6, SIRT7 and histone deacetylase 11 (HDA11), are hypermethylated while HDAC5 and HDAC9 are hypomethylated. We also observed hypermethylation of histone acetylation eraser genes, SIRT6 and SIRT7.

### Methylation patterns of core histone and linker proteins

Chromatin remodeling proteins are complex molecules that can alter the chromatin architecture to enable access to the genomic DNA by transcription regulatory proteins, and allow transcription signals to reach their destinations on the DNA strand. SWI/SNF chromatin remodeling complex proteins ARID1B, SMARCA2 and SMARCD3 are very important for remodeling of chromatin. We observed hypomethylation of CpG sites of SMARCD3, while SMARCA2, ARID1B showed both hyper and hypomethylated CpG sites. A list of all the differentially methylated epigenetic regulatory genes with fold-changes and Δβ values are provided in Supplementary Data 5.

Histone proteins are the primary protein components around which DNA is tightly wrapped. They play essential structural and functional roles in the transition between active and inactive chromatin states, i.e., the euchromatin and heterochromatin. We observed dm-CpGs in core histone protein genes (H2A, H2B, H3 and H4) and the linker protein gene, H1. HIST1H2AG, HIST1H2APS1, HIST1H2BA of core protein H2A are hypomethylated; while in case of H2B, HISTH2BC, HIST1H2NB are hypomethylated and HIST3H2BB, HIST2H2BF, HIST1H2BI are hypermethylated. Similarly, in case of H3 core protein, HIS1H3C, HIST1H3 are hypomethylated and HIST1H3B, HIST1H3E, HIST1H2F, HISTH3G are hypermethylated. However, we did not observe any dm-CpGs in H4 protein genes. The linker protein genes, H1F0 and H1FOO are also hypomethylated in PC (Table 4).

### Differential methylation of homeobox genes

Homeobox-containing genes play vital role in the anatomical development of tissues and organs during early embryonic development stage. It has been reported that these homeobox genes play a vital role in pancreatic cancer cell proliferation [24]. We investigated differential methylation of CpG sites in HOX family and other homeobox-containing genes such as PAX, PRRX, MSX, IRX, SHOX, TGIF, ZEB and HHEX in the TCGA PC data.

**Figure 3:**
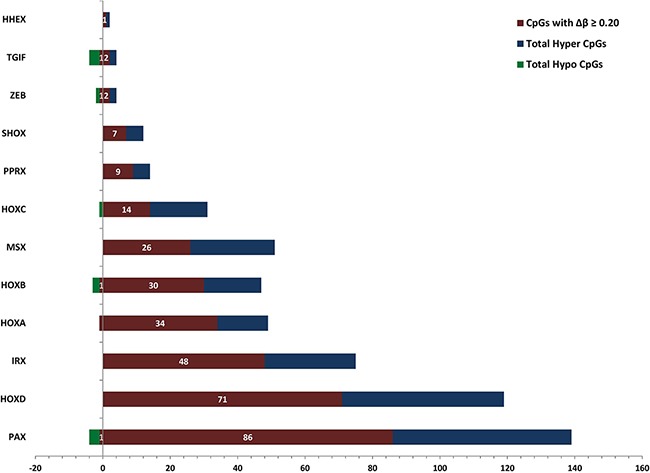
Patterns of DNA methylation in homeobox-containing gene cluster families in pancreatic cancer For each homeobox-containing gene subfamily we calculated the number of differentially hypermethylated and hypomethylated CpG sites that meet the p-value and FDR thresholds of 0.01. Hypermethylated CpGs are on the right side of the Y-axis and hypomethylated on the left side. In the figure, blue and green color shows the total number of differentially hypermethylated and hypomethylated CpGs, respectively, and purple color shows the total number of differentially methylated CpG sites with Δβ ≥ 0.2.

We observed that almost all the dm-CpGs are hypermethylated in HOX family genes except one site in HOXA3 and HOXC4 genes (Supplementary Figure 4 and Supplementary Data 6). Several other homeobox-containing genes also contained dm-CpGs. In the Paired box (PAX) family, a total of eight genes are hypermethylated, while in the case of paired related homeobox (PRRX) gene cluster, only PRRX1 is hypomethylated. In Msh homeobox (MSX) gene cluster, both MSX1 and MSX2 are hypermethylated. An Iroquois homeobox (IRX) family protein, IRX1 is reported as tumor suppressor gene in gastric cancer [25]. IRX1 is not differentially methylated but its homolog IRX2 is hypermethylated in PC. Short stature Homeobox 2 protein (SHOX2) gene hypermethylation, a well-known marker in lung cancer [26] is also hypermethylated in PCs. In case of TGFB-Induced Factor Homeobox (TGIF) cluster proteins; TGIF1 and TGIF2 that are transcriptional repressors of tumor suppressor gene, SMAD are hypomethylated. Zinc Finger E-Box Binding Homeobox (ZEB) cluster protein ZEB1, which is a transcriptional repressor of interleukin-2 (IL-2) gene is hypomethylated, while ZEB2, a repressor of E-cadherin with activated SMAD is hypermethylated. Homeobox-containing gene, HHEX is also hypermethylated in PCs. We observed hypermethylation of most of the HOX-containing genes except the TGIF cluster genes (Figure 3).

### Differential methylation of pancreatic development and pancreatic signaling genes

Panaceas development related genes are activated only at early embryonic development stage, but reactivation of embryonic pancreas development program is common in pancreatic cancer development [27, 28]. Here we looked at the DNA methylation patterns in pancreas development associated genes. We observed that pancreas development and pancreatic signaling related genes are differentially methylated in PCs (Supplementary Data 7). GATA3 [29, 30], FOXA1 [31], neurogenin3 (NEUROG3) [32, 33], NKX2-2, NKX6-1 [32], ISL1, HNF1B, HNF4A, PAX6 [32], HLX, SOX9 [34, 35], MNX1 [36], ONECUT1 [36] are known to play vital role in pancreas development. In pancreatic cancer, GATA3, FOXA1, NEUROG3, NKX2-2, NKX6-1, ISL1, PAX6 and HLX are all hypermethylated. We observed that HNF4A is hypomethylated, while HNF1B, MNX1, SOX9, NKX6-2 and ONECUT1 have both hypermethylated and hypomethylated CpG sites. Homeobox-containing protein, MEIS2 involved in PDX-based pancreas development [37] is also hypermethylated in PCs compared to normal. Matrix metalloproteases 2 (MMP2) and MMP9 are important proteins for pancreas development [38], which are also differentially methylated in PCs.

NOTCH1, NOTCH2, FGF10, HGF, EGF, EGFR are important genes involved in pancreatic signaling; we observed hypermethylation of CpG sites in FGF10 and EGF. Hypomethylation of EGFR, and both hypo and hypermethylation of NOTCH1. We did not observe any dm-CpGs in HGF, but its activator, HGFAC is hypomethylated (Supplementary Data 7).

### Enrichment analysis of differentially methylated genes

Knowing the biological functions of genes containing dm-CpGs is important to understand the effects of methylation patterns of PC methylome. Here, we performed pathway enrichment analysis and gene ontology (GO) enrichment analysis for a total of 4,254 genes that are mapped against dm-CpGs at Δβ≥0.2 by using WEB-based Gene SeT AnaLysis Toolkit (WebGestalt) tool [39]. Out of 4,254 genes, 4,031 are mapped against the Entrez Gene Ids of WebGestalt database. Enrichment of these genes was observed in major KEGG pathways such as pathway in cancer, MAPK signaling pathway, calcium signaling, focal adhesion, regulation of actin cytoskeleton, and gap junction pathways. Table 5 shows the list of top enriched pathways, while complete pathway enrichment results are available in Supplementary Data 8.

**Table 5:** WebGestalt based pathway analysis of differentially methylated genes which have delta beta value more than 0.2 in tumor vs. normal

KEGG pathway	KEGG ID	Ratio	Raw p-value	BH adjusted p-value
Pathways in cancer	05200	3.45	8.36e-31	1.50e-28
MAPK signaling pathway	04010	3.55	2.06e-27	1.84e-25
Neuroactive ligand-receptor interaction	04080	3.50	6.95e-27	4.15e-25
Calcium signaling pathway	04020	4.17	2.98e-26	1.33e-24
Focal adhesion	04510	3.91	1.33e-25	4.76e-24
Regulation of actin cytoskeleton	04810	3.72	2.11e-24	6.29e-23
Endocytosis	04144	3.35	2.12e-18	5.42e-17
Metabolic pathways	01100	1.76	1.63e-14	2.92e-13
Vascular smooth muscle contraction	04270	3.69	9.39e-14	1.53e-12
Axon guidance	04360	3.48	2.16e-13	3.22e-12
ECM-receptor interaction	04512	4.15	2.87e-13	3.95e-12
Leukocyte transendothelial migration	04670	3.60	4.81e-13	6.09e-12
Pancreatic secretion	04972	3.71	2.73e-12	3.05e-11

Jones *et. al*. provided a list of core signaling pathways and processes that are altered in most of the PCs [40]. Here we observed enrichment of several of those core signaling pathways such as apoptosis, hedgehog signaling, TGF-β signaling, Wnt signaling, Notch signaling and cell cycle pathways. Enrichment of genes involved in pancreatic cancer pathway and pancreatic secretion was also observed. Apart from these core PC pathways, we also observed enrichment of several other important pathways that are related to cancer such as p53 signaling, VEGF signaling, phosphatidylinositol signaling, EbrB signaling, Jak-STAT signaling, Fc gamma R-mediated phagocytosis, insulin signaling, cytokine-cytokine receptor interaction, chemokine signaling pathway, natural killer cell mediated cytotoxicity, T cell receptor signaling pathway and B cell receptor signaling pathway. Enrichment of tight junction, cell adhesion molecules, adherens junction pathways was also observed in PC (Supplementary Data 8). These enriched pathways suggest that apoptosis, cell-cycle/cell differentiation, cytoskeleton structure, immune response, DNA damage responses are highly affected by DNA methylation in PC.

Using Ingenuity Pathway Analysis (IPA), we identified that axonal guidance signaling, G-protein coupled receptor signaling, hepatic fibrosis/hepatic stellate cell activation, molecular mechanism in cancer, Sertoli cell-Sertolli cell junction are top enriched canonical pathways. We also observed the enrichment of several other canonical pathways such as TGF-β signaling, HGF signaling, apoptosis signaling, cell adhesion and Wnt pathway, which are also important in pancreatic cancer (Supplementary Data 9).

To determine the functional relevance of differentially methylated genes, we also carried out GO enrichment analysis using WebGestalt. GO terms related to cell division and cell differentiation are in the enriched list of GO biological function. Other enriched GO terms include system development, developmental process, nervous system development, anatomical structure morphogenesis, cell differentiation, skeletal system development, cell-cell signaling (Supplementary Data 10). We also used DAVID [41] in R for pathways and GO enrichment analysis. DAVID based pathway and GO enrichment analysis also showed similar results at FDR 0.05 (Supplementary Figure 5).

### Clustering analysis using differential methylation data

**Figure 4:**
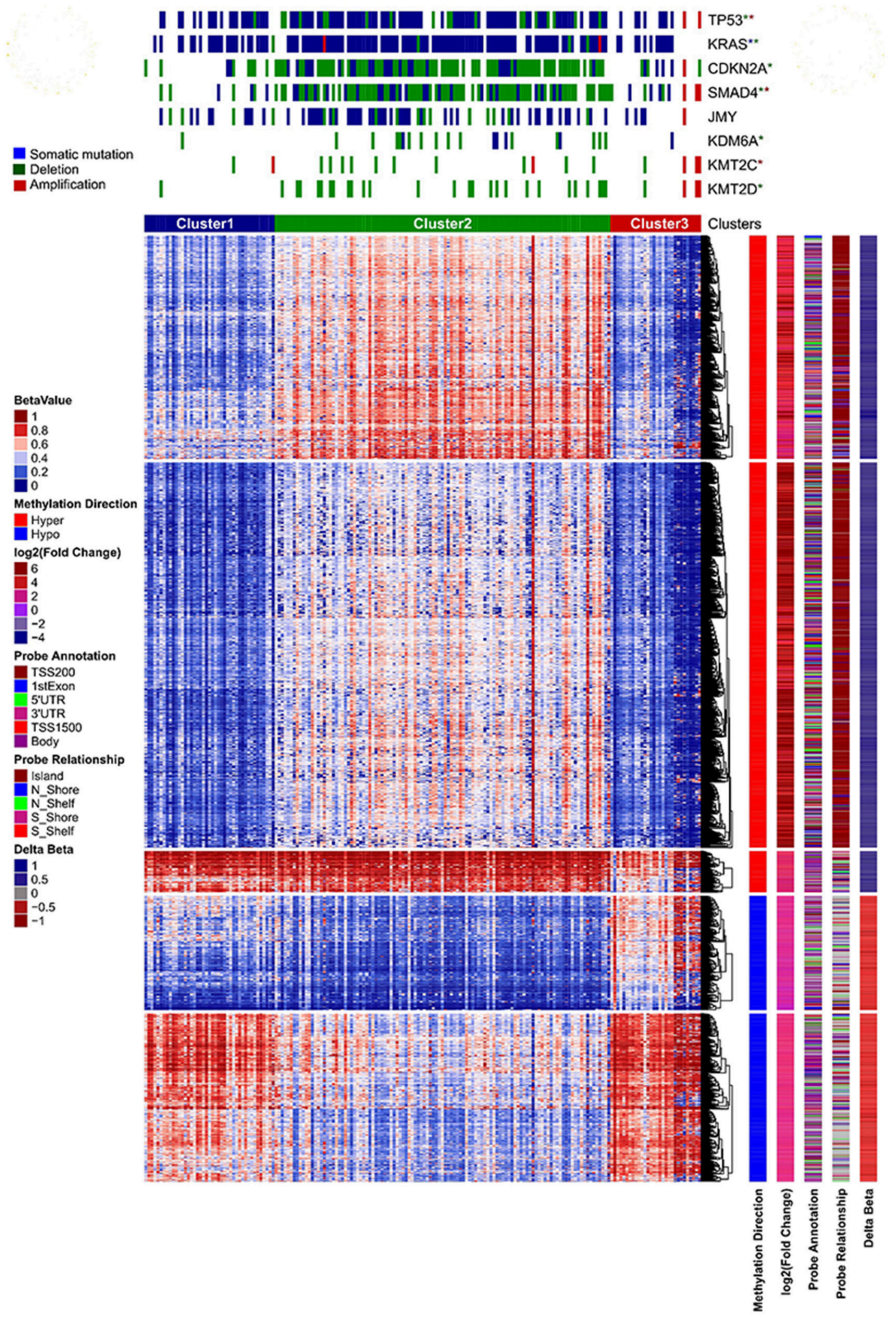
Unsupervised clustering of PC patient data on the basis of differentially methylated CpG sites We used only the CpG sites with Δβ ≥ 3. We used NMF for consensus clustering of samples by using 500 permutations. Vertical sidebars show information on the direction of methylation, fold change in tumor, probe relationship and probe annotation of differentially methylated CpG sites. Top annotations of heatmap plot are somatic mutations, copy number deletion and amplification from cBioPortal.

Identifying clinically relevant subtypes of a cancer based on the DNA methylation patterns is an important computational problem in medicine, which helps to make an assertion to provide specific and efficient treatment options for patients of different subtypes. Unsupervised hierarchical clustering analysis based on the methylation patterns of PC patients is a very effective way to identify defined patient sub-groups. Here, we first clustered by the patient samples and then by the CpG sites to generate both patient clusters (columns) and CpG clusters (rows) (Figure 4). We used a stringent β value (Δβ ≥ 0.3) of 2,751 CpG sites for unsupervised clustering using non-negative matrix factorization (NMF) [42] method. We defined the number of clusters from 2 to 7 with 500 iterations each to determine the optimum number of clusters. We observed that three patient clusters are optimal as they showed the highest cophenetic constant, 0.994 with average silhouette width of 0.72 (Supplementary Figure 6). Copenhetic constant, which ranges from −1 to +1 is used as a measure of the reproducibility of clustering results; positive scores mean good clustering. A second measure, Silhouette width (also ranges from −1 to +1) indicates how good the data are grouped in clusters, positive scores mean data are appropriately clustered. The three patient clusters showed very distinct patterns of hyper and hypomethylated CpGs. We used the Bioconductor tool *ComplexHeatmap* [43, 44] for heatmap analysis of clustering. We also did clustering of CpG sites (row-wise) using k-means clustering in five clusters. As shown in Figure 4, CpG clusters 1-3 are hypermethylated and clusters 4 and 5 are hypomethylated in PC.

We downloaded somatic mutation and copy number data from cBioPortal [45] and did enrichment analysis of important oncogenes and tumor suppressor genes in these three patient clusters, using Fisher's exact test with a p-value cutoff of 0.05. Patient cluster two exhibited the most distinctive patterns of genome alterations (Figure 4). TP53, KRAS and JMY are highly mutated in cluster 2 compared to others. Similarly, high copy number deletion of CDKN2A, SMAD4, KDM6A and KMT2C and KMT2D genes (P < 0.05) was noticed in cluster 2. On the other hand, cluster 3 exhibited high amplification of TP53, SMAD4, JMY, KMT2C and KMT2C genes (P < 0.05) (Figure 4). We further carried out analysis to correlate the patient clusters with their clinical data on tumor grade and stage and identified significant relationships. Neoplasm histological grade G1 (P 3.44e-05) and pathologic T-stage T2 (P 0.0059) are enriched in patient cluster 3. Cluster 2 showed enrichment of pathologic T-stage T3 (0.0194), while cluster 1 has neoplasm histological grade G2 (P 0.045) enrichment. We did not observe enrichment of gender, age, race, or ethnicity in any cluster.

### Differential gene expression analysis

We used *edgeR*, which is one of the best tools for differential gene expression analysis for RNASeq data [46]. We observed a total of 258 differentially expressed genes (DEGs) with both p-value and adjusted p-value below 0.05 and a minimum logFC value of two (Supplementary Data 11). Of these, 27 are up-regulated and 231 are down-regulated in PC samples compared to normal samples (Supplementary Figure 7).

We used log2 transformed expected counts data of DEGs for clustering using Ward's distance measure for hierarchical clustering using the ComplexHeatmap package. We observed two major clusters, but these clusters are not as sharply distinguishable as DNA methylation clusters (Supplementary Figure 8). We also did enrichment analysis of DEGs using WebGestalt and IPA. We observed enrichment of hematopoietic cell lineage, cytokine-cytokine receptor interaction, B cell receptor signaling pathway, natural killer cell mediated cytotoxicity, chemokine signaling pathway (Table 6). Apart from these pathways, we also observed enrichment of neuroactive ligand-receptor interaction, leukocyte transendothelial migration, and intestinal immune network for IgA production in PC (Supplementary Data 12).

**Table 6:** WebGestalt based pathway enrichment analysis of differentially expressed genes in TCGA pancreatic cancer data

KEGG pathway	KEGG ID	Ratio	Raw p-value	BH adjusted p-value
Hematopoietic cell lineage	4640	19.84	9.65e-11	2.22e-09
Primary immunodeficiency	5340	34.92	1.09e-09	1.67e-08
Cytokine-cytokine receptor interaction	4060	8.57	5.50e-09	6.33e-08
B cell receptor signaling pathway	4662	18.62	1.25e-08	1.15e-07
Natural killer cell mediated cytotoxicity	4650	11.55	1.02e-07	7.82e-07
Chemokine signaling pathway	4062	9.24	1.62e-07	1.06e-06
Neuroactive ligand-receptor interaction	4080	6.42	4.41e-06	2.25e-05
Leukocyte transendothelial migration	4670	7.53	0.0006	0.0023
Graft-versus-host disease	5332	12.78	0.0017	0.0056
Intestinal immune network for IgA production	4672	10.91	0.0027	0.0083
Autoimmune thyroid disease	5320	10.07	0.0033	0.0095
Cell adhesion molecules (CAMs)	4514	5.25	0.0074	0.0197
Antigen processing and presentation	4612	6.89	0.0096	0.0232

When we used IPA for canonical pathway analysis of DEGs, we observed primary immunodeficiency signaling, altered T cell and B cell signaling, crosstalk between dendritic cell and natural killer cells, PI3K signaling in B lymphocytes, B cell development and natural killer cell signaling as major enriched pathways (Supplementary Data 13). We also observed IRF4, BCR (complex), IL12 (complex), SATB1 and BL6 as the most significantly activated upstream regulators in PC. These upstream regulator genes are involved in differentiation, apoptosis, and proliferation.

Biological significance of DEGs is determined by GO enrichment analysis using WebGestalt. We observed GO enrichment of immune response, immune cell activation, and immune cell differentiation in PC. Enriched functions include cell-to-cell interaction, cellular development, cellular growth and proliferation, super oxide generation and molecular transport with adjusted P-value 0.05 (Supplementary Data 14). We observed the enrichment of similar pathways and ontologies at FDR 0.01 by using DAVID [41] in R package (Supplementary Figure 9).

### Correlation of DNA methylation and gene expression

Correlation between DNA methylation and gene expression was determined to estimate to what extent gene expression may be influenced by DNA methylation in pancreatic cancer. We calculated expression quantitative trait loci (eQTL) based on non-zero Pearson correlation between gene expression and DNA methylation levels of CpG sites within 100 kb of corresponding gene's TSS by using linear regression (eMap1 function) in an R based tool, *eMap* [47]. The eQTL analysis allows us to determine the locus of the genome (eQTL) containing variation in DNA methylation that influences the expression levels of one or more genes. Any association is considered as significant if the Bonferroni corrected p-value is less than 0.05. We observed that a total of 21,519 CpG sites were significantly correlated with the expression of 4,565 genes in PC (Supplementary Data 15).

**Figure 5:**
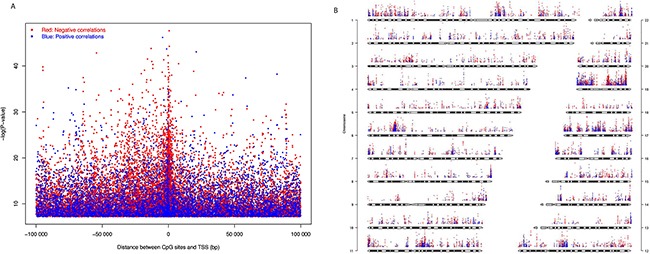
CpG sites whose DNA methylation levels were significantly correlated with gene expression with Bonferroni corrected P-value < 0.05 **(A)** Significance level of correlation between DNA methylation β value and gene expression plotted against distance between CpG sites and transcription start site (TSS). **(B)** Significance level and genome-wide distribution of correlation between DNA methylation and gene expression. Red dots represent negative correlation and blue dots represent positive correlation. We did not use sex chromosomes in this analysis.

The expression levels of 2,298 genes are positively correlated with the DNA methylation level of at least one CpG site, i.e., higher the methylation, higher the gene expression, while the expression level of 3,657 genes are negatively correlated with the methylation level of at least one CpG sites. Positive correlated CpG sites were quite evenly distributed at both up and downstream of TSS (Figure 5A). Negative correlated CpG sites were also found at both up and downstream of TSS (Figure 5A), but they were enriched close to the TSS (1500 bp up-stream to 5,000 bp downstream). The CpG sites that correlated with gene expression were distributed across the whole genome, but chromosomes 1, 6p, 11, 12, 16, 17, 19 are highly enriched (Figure 5B).

**Figure 6:**
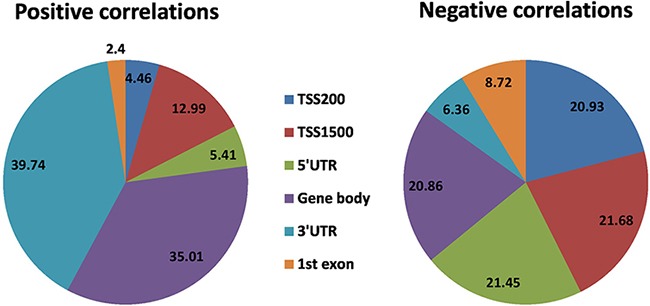
Significant correlation between DNA methylation patterns in different gene regions and gene expression (Bonferroni corrected P-value < 0.05) Pie chart plot shows the distribution of negative and positive correlations corresponding to the functional regions of genes. Distribution patterns are very different for the positive correlations compare to negative correlations.

Two-sided non-zero Pearson correlation was also calculated between gene expression and DNA methylation of corresponding ‘gene subregions’ by using the R function, *cor.test*. The expression of a total of 7,882 genes significantly correlated with DNA methylation level in at least one gene region (Supplementary Data 16). Of these, the expression of 6,088 genes is negatively correlated with the methylation level of CpG sites, and the expression of 1,794 genes is positively correlated. A combined ~43% of negatively correlated genes have methylated CpGs within 1,500bp upstream of TSS (TSS200 and TSS1500), while only ~17% genes show positive correlation with methylation in the same region. On the other hand, 5′UTR showed ~21% negative correlations, while only ~5% positive correlations in the same region. First-exon has only ~9% and ~2% positive and negative correlations, respectively. Of the positive correlations, around ~40% were found in the 3′UTR and ~35% found in the gene body, while only ~25% in remaining sub-regions. It means around ~83% positive correlations were found outside of the promoter regions (TSS200 and TSS1500), while ~64% negative correlation were found in upstream regulatory region i.e. promoter plus 5′UTR (Figure 6). In summary, differential methylation in the upstream regulatory regions resulted in more negative correlations with gene expression while in the downstream regions differential methylation resulted in more positive correlation with gene expression (Supplementary Figure 10). We also observed that methylation in 22 homeobox-containing, 34 epigenetic regulatory, nine histone protein and three chromatin remodeler protein expressions are also correlated with gene expression (Supplementary Data 17).

### Epigenetic-driven gene analysis

We used MethylMix [48, 49] to identify epigenetic-driven genes, which are hyper or hypomethylated in cancer and also have significant predictive effect on gene expression. Any gene with an adjusted p-value of 0.001 between DNA methylation and gene expression correlation is considered as an epigenetic-driven gene. In this study, we identified 11 genes (CARD16, DHRS2, FGL2, GBP2, LOC541471, LRRIQ1, NCRNA00152, PTPLAD2, PTX3, SPATA12 and TST) as potentially epigenetic-driven. Of these, FGL2, GBP2, LOC541471, LRRIQ1, PTPLAD2 are hypermethylated, DHRS2, NCRNA00152, PTX3, SPATA12, TST are hypomethylated and CARD16 is both hyper and hypomethylated genes.

### Regulatory element landscape and transcription factor analysis

We investigated the coherence between PC-specific hypermethylated and hypomethylated CpG sites and known transcription factor (TF) binding sequence motifs to get details about which site-specific TFs may be involved in regulating the PC specific DNA methylation pattern. We used ELMER to identify differentially methylated distal enhancer probes and then the upstream regulators of methylation patterns.

For differential methylation analysis of distal enhancer region we used both the cut-off p-value and BH adjusted p-value at 0.01. A total of 1,744 distal enhancer probes are significantly hypermethylated and 4,306 are significantly hypomethylated in PC. To get high confidence results for distal enhancer probe and gene expression correlations, we used 10,000 permutations. In case of hypermethylated, 914 distal enhancer probes showed statistically significant correlation with gene expression (Supplementary Data 18). A total of 91 JASPAR and Factorbook TF binding sequence motifs were enriched with at least 10 statistically significant hypermethylated probes. Finally, we compared the average DNA methylation of all distal enhancer probes within +/− 100bp of an enriched motif with the expression of 1,982 known human TF genes [50]. We found 25 TF binding motifs whose DNA methylation has statistically significant association with TF gene expression (Supplementary Data 19). All TFs that fall in the top 5% of known motif-TF pair ranking are considered as candidate upstream regulator of distal enhancer probe hypermethylation. Similarly in case of hypomethylation, we observed that 1,492 probe-gene pairs have statistically significant correlation (Supplementary Data 18). These 1,492 probes are enriched in 91 JASPAR and Factorbook TF binding sequence motifs with a minimum of 10 significantly hypomethylated probes in each TF motif enrichment region. We also observed that 6 TF binding motifs have statistically significant association with known TF gene expression. Our analysis suggests that several HOX, SOX, NKX and SMAD genes are the upstream regulators of methylation of distal enhancer probes (Supplementary Data 19).

## DISCUSSION

DNA methylation is an important regulator of transcription, and its role in oncogenesis has been a topic of interest in understanding cancer biology. Alterations in DNA methylation are commonly found in a variety of tumors and global DNA methylation has been recognized as a causative factor of oncogenesis [51, 52]. It has been well known that subtle differences in the DNA methylation patterns could significantly affect gene expression and this information could be used as a biomarker to distinguish the cancerous cells from normal cells [53–55]. Previous reports from Sato *et. al*. [14] and Tan *et. al*. [15] showed DNA methylation patterns from pancreatic cancer. However to our knowledge, exhaustive analysis of the global pancreatic cancer DNA methylation data from TCGA has not been reported. Sato *et. al*. used methylation-site specific PCR for methylation analysis, while Tan *et. al*. used GoldenGate methylation cancer panel array that has low genome coverage and low sensitivity when compared to the HumanMethylation450 BeadChip used by TCGA. Apart from the smaller data size, these studies have used formalin-fixed paraffin embedded samples, xenografts and pancreatic cancer cell lines that may have limitations on the quality of data generated from their experiments. Our study on the other hand has used TCGA data from the Illumina HumanMethylation450 chip obtained from fresh tissue samples, which has very high genome coverage with higher accuracy and consistency. Here we carried out comprehensive analysis of global differential methylation, differential gene expression, and correlated the methylation and gene expression data. To ignore gender bias we removed all the CpG probe and gene expression data from X and Y chromosomes from our analysis.

We studied the global methylation patterns of pancreatic cancer across the genome and observed that all chromosomes have differentially methylated CpG sites (dm-CpGs) (Figure 1A). Differential methylation analysis suggests that while dm-CpGs are distributed across the entire genome, chromosomes 4, 5, 13, and 18 are predominantly hypermethylated and chromosome 17 is predominantly hypomethylated. Length normalized results show that chromosome 19 has the highest methylation frequency and chromosome 9 has the lowest (Figure 1B). CpG islands and regions in close proximity to CpG island (shores and shelfs) have more hypermethylated CpG sites compared to the regions which are far away from islands (Supplementary Data 3). CpG island analysis suggests that most of the CpG islands are hypermethylated (Figure 2). When we calculated the correlation between gene expression and DNA methylation of CpG sites between 100kb of TSS, we observed that negatively correlated CpG sites (hypermethylation of CpGs resulting in reduced gene expression) are predominantly located closer to TSS in the upstream region, while positively correlated ones are in the down-stream region to the TSS (Figure 5A). This result corroborates the key regulatory role of DNA methylation changes in the promoter region on gene expression compared to non-promoter regions. When we look at different gene sub-regions, around 75% of the positive correlations between DNA methylation and gene expression levels were found in the gene body and 3′UTR (non-promoter regions). Similar observations were also found in other cancer types that include chronic lymphocytic leukemia [56] and breast cancer [57]. On the other hand, around 64% upstream CpG sites (i.e. TSS200, TSS1500 and 5′UTR) are negatively correlated with gene expression which supports that promoter hypermethylation is an important alternative biological phenomenon for gene silencing (Figure 6). We also observed a total of eleven epigenetic-driven genes in TCGA PC samples. The positive relationship between gene body DNA methylation and gene expression was previously reported [58]. But role of non-promoter hypermethylation is not well understood; it was hypothesized that it might play an important role in nucleosome positioning, modulation of chromatin structure, enabling enhancer region availability and gene body regulation of alternative promoter [59]. It is also possible that due to the strict parameters we used for statistical significance determination, we might have underestimated the association between DNA methylation and gene expression in the non-promoter region.

Epigenetic regulatory pathway genes are differentially methylated in pancreatic cancer patients. DNA methyl transferases (DNMTs) play a very important role in DNA methylation in cells [60–62] and we observed that DNMT1, DNMT3A and DNMT3B are themselves differentially methylated in pancreatic cancer. A very recent research suggests that TP53 interacts with H3K4 histone methyl transferase-2 (MLL2), MLL3 and MOZ genes and play major role in chromatin regulation [63, 64]. We observed differential methylation of MLL2, MLL3, and also H3K9 N-methyl transferases EHMT1 in PC. Although the epigenetic writer gene, lysine acetyltransferase 6A (KAT6A/MOZ) is not differentially methylated, the other KAT proteins such as KAT6B and KAT2A are differentially methylated in PC. Histone-lysine N-methyltransferases SETDM3, STDM7, SMYD2, SMYD3, EZH2 genes are also differentially methylated. Differential methylation of these writer genes indicates their potential role in altering the dynamics of transcription in PC. Apart from these, we also observed dm-CpGs in components of chromatin regulator SWI/SNF complex, core histone proteins and linker proteins. Methylation of these epigenetic regulatory pathway genes can affect global methylation patterns in pancreatic cancer.

Previous reports on transgenic mice suggest that histological changes and genetic aberrations in pancreatic cancer development are similar to embryonic pancreas development [27, 65]. PDX1 is the most important gene for the development of pancreas in early embryonic stage but we did not observe any dm-CpG sites in this gene. However, several other genes e.g. GATA, HNF1A1/4A, ONECUT1, MNX1, NKX2.2/6.1/6.2, NEUROG3, PAX6, FOXA1 that play major role in pancreatic development are differentially methylated. Matrix metalloproteases (MMPs) are required for branching morphogenesis of several organs in pancreatic development. MMP2, MMP9 are two important MMPs that play a vital role in pancreas development got differentially methylated in pancreatic cancer. Pathway enrichment analysis suggests that major pancreatic development signaling pathways, i.e., Hedgehog, Notch and TGF-β related genes are affected by differential methylation. We also observed differential methylation of RMRM (reprimo), CLDN5, LHX1, NTPX2, SPARC and ST14, which are already reported as aberrantly methylated genes in pancreatic adenocarcinoma [14].

SMAD proteins are important components of TGF-β signaling pathway and play vital role in pancreatic cancer patient's survival by causing cell cycle arrest at the G1 phase [66–68]. TGIF1 and TGIF2 that are repressors of tumor suppressor SMAD proteins are also differentially methylated. We also observed differential methylation of several repressors of IL-2 and E-cadherin proteins. It is a well-known fact that GATA3 expression is associated with disease free survival and can reverse the cancer metastasis [69]. We did not observe statistically significant changes at gene expression level in GATA3, but we observed hypermethylation of 13 CpG sites in PC. GATA4, recently reported as a major player in connecting autophagy and DNA damage response to senescence and age dependent inflammation [70] is also differentially methylated in PC.

Homeobox containing genes are important for the development of tissues and organs in human. We observed differential methylation of several important homeobox proteins (Figure 3) e.g. HOXA1, HOXA2, HOXB1, HOXB3, HOXB7, HOXC4, HOXC9, HOXD4, HOXD8, HOXD10, HOXD11, HOXD12 and HOXD3 (Supplementary Figure 4). We also observed differential methylation of several other genes containing homeobox; e.g. PAX1, PAX2, PAX3, PAX6, PAX8, PAX9, PRRX1, SHOX2, MSX1, MSX2, IRX1, SATB, HHEX, CDX and LHX. TGIF1, a suppressor of SMAD and an important homeobox contacting protein that plays a vital role in pancreatic cancer patient's survival is also differentially methylated (Supplementary Figure 4). ZEB1 and ZEB2, important transcription regulators that interact with SMAD are also differentially methylated in PC. We also observed the differential methylation of four pancreatic cancer marker genes, i.e., FOSB, KLF6, ATP4A, GSG1, related to survival of patients [71].

Pathway enrichment analysis of differentially methylated genes covers major pathways related to cell division, differentiation, migration and other biological processes, which are very important in cancer development and progression. Core pancreatic cancer related signaling pathways e.g. Wnt, Notch, TGF-β, Hedgehog, apoptosis and cell cycle [40] were affected by DNA methylation in our study. Axon guidance pathway was also enriched in IPA and WebGestalt analyses, which was also reported as a major affected pathway in pancreatic cancer [72]. Pancreatic cancer pathway (hsa05121) also got affected with DNA methylation.

We observed differential expression of 258 genes in PC, pathway enrichment analysis of DEGs suggest that immune system is the most affected. From the cytoband analysis of DEGs using WebGestalt, we observed the enrichment of chromosome arm 19q and 6p (Supplementary Data 20). We observed that a number of dm-CpG sites are also enriched on those cytoband, which are also enriched with genes that exhibit significant correlation between DNA methylation and gene expression (Figure 5B). Our results suggest that cell division and cell differentiation are major affected pathways, but infiltration of immune cell might be taking place because we observed that T-Cell and B-cell proliferation and activation related pathways are also affected.

Distal enhancer and transcription factor analysis suggest that several important TFs are upstream regulators in pancreatic cancer. We observed that 25 TF binding motifs are affected by DNA hypermethylation, while only six by hypomethylation in PCs. HOXA and HOXB TFs are upstream regulators in distal enhancer hypomethylation, while only the HOXD8 in enhancer hypermethylation. SMAD3 and SMAD6 are upstream regulators in enhancer hypomethylation while only SMAD4 in enhancer hypermethylation. SOX11 and SOX21 are hypomethylated upstream regulators while SOX17 is the upstream regulator of hypermethylation. We observed that zinc finger proteins (except ZNF488), NKX2-3, NKX6-1, STAT4 and STAT5B are also upstream regulators of enhancer hypermethylation.

Recently, Waddell *et. al*. [2] proposed molecular sub-types of pancreatic cancer on the basis of point mutation and structural variation using PC data from ICGC resource. Most recently, Bailey *et. al*. [73] also proposed a new method for molecular sub-typing of PC using ICGC data. Also, Waddell *et. al*. proposed a method based on structural variation and somatic mutation data; however, this method work only when we have whole genome sequencing data. On the other hand, Bailey *et. al*. proposed an integrative method using the whole exome, whole genome, whole transcriptome and microarray data. Apart from these, Bailey et. al. used the top 2,000 most variable genes for clustering analysis rather than differentially expressed genes. Both the methods need either high depth whole genome sequencing or more than one type of data for subtyping. Here we performed molecular sub typing of PC solely based on DNA methylation. Our clustering analysis based on β value of dm-CpG sites suggests that there are three possible sub-groups in TCGA PC (Figure 4). These clustering sub-groups exhibited different somatic mutation and copy number alteration patterns. Neoplasm histological grade G2 is enriched in cluster 1 and pathologic T-stage T3 is enriched in cluster 2. Cluster 3 has enrichment of neoplasm histological grade G1. However, clustering based on gene expression was not informative in our study as we did not observe distinctive clustering patterns using DEGs (Supplementary Figure 8).

## CONCLUSIONS

DNA methylome of pancreatic cancer shows significant changes compare to normal pancreatic tissues. To our knowledge, this is the first global DNA methylation analysis of TCGA PC data on PC patients. We observed differential methylation of FOSB, KLF6, ATP4A, and GSG1 genes that are previously reported as markers for pancreatic cancer survival. Our clustering analysis based on methylation patterns suggests that there are three potential sub-types of PC in TCGA. These clusters showed enrichment of histological grade, pathological grade and common PC genomic aberrations. Furthermore, our analysis shows that major signaling pathways related to pancreatic cancer and pancreas development are perturbed with DNA methylation. We observed the enrichment of all major pathways as cell adhesion, hedgehog, TGF-β, Wnt, Notch which was also reported by Nones *et. al*. from ICGC PC data. Epigenetic signaling pathway related genes also got affected with DNA methylation in pancreatic adenocarcinoma. Correlation, both positive and negative, between DNA methylation and gene expression was observed in substantial part of the genome. Upstream CpG sites close to TSS showed mostly negative correlations confirming the regulatory role of epigenetic changes in this region on gene expression. HOX cluster proteins, SOX11, SOX21 and histone core proteins are upstream regulators of enhancer hypomethylation, and zinc finger proteins (ZNF), SMAD4, STAT4, STAT5B, NKX2-3 and NKX6-1 are upstream regulators of enhancer hypermethylation. Pathway enrichment analysis of differentially methylated and DEGs suggests that immune system related pathways are top affected pathways suggesting that immune cell infiltration is taking place in the tumors.

## MATERIALS AND METHODS

### DNA methylation and RNAseq data

We downloaded TCGA Firehose level-3 data for DNA methylation and gene expression using Bioconductor tool RTCGAToolbox [74]. We used the Illumina HumanMethylation450 BeadArray data for DNA methylation analysis; and IlluminaHiSeq RNASeqV2 data for gene expression analysis. The DNA methylation level-3 data contain β values for 485,578 CpG sites with annotations for HUGO Gene Nomenclature Committee (HGNC) gene symbols; chromosomes (UCSC hg19) and CpG coordinates (UCSC hg19). These β values calculated as (M/M+U) range from 0 to 1, where M is methylated allele frequencies and U is unmethylated allele frequencies; so higher β values indicate higher methylation. The gene expression data were obtained as single RSEM (RNAseq by Expectation Maximization) values for 20,531 HGNC genes.

### Methylation data processing

We removed β value for those CpG probes that were either mapped against chromosomes X and Y to remove gender biases, or missing in more than 20% of the samples. We also used *k* nearest neighbor based imputation to estimate the remaining missing values in the data [75]. Statistical analyses of DNA methylation of 194 samples (184 primary tumors and 10 normal samples) were performed at two different levels, i.e. the CpG site level and the gene level. For distal enhancer analysis, we used probes that are 2kb away from the TSS in the *cis*-region.

We separately analyzed the CpG probes that are mapped to genes in six different subregions: TSS200 (region from TSS to − 200 bp upstream of TSS), TSS1500 (200-1,500 bp upstream from TSS), 1^st^ exon, 3′UTR, 5′UTR and gene body. We also analyzed DNA methylation in UCSC CpG island, shores (regions 0–2 kb from CpG islands), shelves (regions 2–4 kb from CpG islands), and open sea regions (CpG sites in the genome that do not have a specific designation). The ‘gene region collapsed data’ were constructed to reduce the dimensionality of methylation data on regions that are most relevant for gene function. DNA methylation levels for each sub-region were summarized using the median, i.e. if a gene has more than one CpG site in the same subregion we used the median of β values (by using ‘aggregate’ function in R). We also did differential methylation analysis at CpG Island level. For dm-CpG island analysis, we used median of β value of all CpG sites in the known UCSC island. Only those CpG islands that have at least three CpG sites with β value after preprocessing steps were used for differential CpG island analysis.

### RNASeq data processing

The level-3 RNASeq data have gene level expression value, meaning any alternate isoforms are included in a single normalized RSEM expression value. These values were derived by mapping RNASeq reads with MapSplice and quantifying with RSEM [76]. The level-3 data in TCGA has different types of data for RSEM, but we used the expected count data in this analysis. We removed all samples that lack expression values for more than 20% of the genes, and also removed genes that lack expression values for which more than 20% of the samples from our analyses. We used the *k* nearest neighbor imputation for handling missing expression values in the data.

### Differential methylation analysis

We removed all those probeshaving a SNP within 10bp of interrogated CpG sites as suggested by previous TCGA studies [77, 78]. We also removed the probes on chromosomes X and Y and those in the repeated regions of chromosomes. We calculated β values for CpGs, which have missing β value in ≤ 20% samples by using 15-nearest neighbor in imputeKNN module of R tool, *impute*. We used the R package, “*samr*” [79] with 10,000 permutations for differential methylation analysis. For a CpG site to be considered differentially methylated, the difference in the median β value in primary tumor and normal samples should be at least 0.1 and the FDR q-value should be less than 0.01. We also used the same parameter in *samr* for differential CpG island analysis on summarized methylation data. Then, we calculated the methylation frequency per mega base pairs (Mb) for each chromosome. For this, we calculated the total number of methylation for each chromosome and divided by the chromosome length (Mb). Similarly, we also calculated hypermethylation and hypomethylation frequency for each chromosome. If the ratio between hypermethylation to hypomethylation frequencies is ≥ 1.5, we consider that a particular chromosome is predominantly hypermethylated. Similarly if hypomethylation to hypermethylation frequency ratio is ≥ 1.5 we consider that chromosome as predominately hypomethylated.

### Differential gene expression analysis

For differential gene expression analysis, we used the expected counts data from 178 primary and 4 normal samples. We used the Bioconductor tool, *edgeR* [80] for differential gene expression analysis. Similar to DNA methylation, we calculated missing gene expression values using 15-nearest neighbor in imputeKNN option in R tool, *impute*. We used a cutoff value of 0.05 for both raw p-value and Benjamini-Hochberg (BH) adjusted p-value for differential expression analysis.

### Correlation between DNA methylation and gene expression

For correlation analysis, we used 178 primary tumor samples that contain both DNA methylation and gene expression data. Correlation between DNA methylation and gene expression was done by using linear regression in R package, *eMap* [47]. Methylation and expression levels of genes were tested for non-zero correlation using Pearson's correlation i.e. exclude all those with a correlation value of zero. For analysis, we used probes within 100 kb of TSS of a gene, an association was considered as significant if Bonferroni corrected p-value was less than 0.05. Genome wide correlation between DNA methylation and gene expression was visualized using R package, *quantsmooth* [81].

The median methylation of CpGs in the region of ‘gene region collapsed’ and gene expression of corresponding gene was tested for non-zero correlation using Person correlation (R function cor.test). Correlation between DNA methylation and gene expression was considered as significant if the raw p-value and BH corrected p-value were both less than 0.05.

### Epigenetic-driven gene analysis

DNA methylation level of a gene has a significant effect on its corresponding gene expression to be considered as epigenetic-driven gene. For epigenetic-driven gene analysis we used Bioconductor tool MethylMix [48, 49, 82]. First, each CpG sites is associated with its closest corresponding genes. Next, MethylMix check the effect of DNA methylation on its corresponding gene expression in order to be considered as epigenetic-driven gene. MethylMix is computationally very exhaustive tool; it runs for each CpG and corresponding gene pair in parallel mode. Many CpGs show correlated methylation profiles, therefore to improve the speed we used hierarchical clustering to create cluster of CpGs with similar methylation profiles. We used 1-pearson correlation as a distance with cutoff correlation 0.3 in each cluster to define CpG clusters. Then we summarize the CpG-cluster by taking the average β of all CpGs in that cluster. First, we used an adjusted p-value cutoff of 0.05 and delta β cutoff of 0.10 for differentially methylated CpGs or CpG-clusters. Later we used linear regression to model the expression of each gene in cancer with its DNA methylation β values. We used cutoff p-value of 0.001 and negative correlation between DNA methylation and corresponding gene expression to select epigenetic-driven genes.

### Clustering analysis

Non-negative matrix factorization (NMF) was used to identify optimum number of clusters in PC methylation data. We used the R tool, *NMF v-0.20.6* [83] for clustering, and *ComplexHeatmap* [43, 44] for generating heatmap plots. Differentially methylated CpG sites with Δβ ≥ 0.3 were used as input in NMF. NMF parameters include Burnet algorithm, k=2 to k=7 clustering and number of iterations equal to 500. The preferred clustering result was determined by using the observed cophenetic correlation coefficients between clusters and the average silhouette width of consensus cluster members by using R package, ‘*cluster’*. Fisher's exact test was used in R to evaluate the enrichment of genes in clusters.

### Enrichment analysis

We used an online tool, WebGestalt for enrichment analysis of differentially methylated and DEGs [39]. HGNC genes symbols were uploaded and analysis was performed against human reference genome using a BH multiple adjustment threshold of 0.01 and a minimum number of four genes per category. We also used Ingenuity Pathway Analysis (IPA) for canonical pathway and upstream regulator analyses. In case of IPA analysis, we used only the human pathway data knowledge base at a BH multiple correction p-value 0.01 of Fisher exact test.

### Regulatory element landscape and transcription factor analysis

For regulatory element analysis we used the Bioconductor tool, *ELMER* (Enhancer Linking by Methylation/Expression Relationship) [84]. In ELMER, we used level-3 data from tumor samples that have both DNA methylation and gene expression data and control samples DNA methylation data. *ELMER* used ENCODE/REMC ChromHMM, FANTOM5 genomic region as annotated enhancer region. We used *ELMER* to find out the distal enhancer probes (in known enhancer region and >2.0 kb away from known TSS by UCSC gene annotation) and correlates enhancer state with expression of nearby genes to identify transcriptional target. In the first step, we will find out distal enhancer probes and used one tailed t-test to find out hypermethylated and hypomethylated distal probes. In the next step, we determined the correlation between the differentially methylated distal enhancer probe and 10 nearest up or downward gene expression values to find out putative target gene and distal enhancer probe pair. Further, *ELMER* will find the enriched TF binding motifs for differentially methylated distal enhancer probes which are significantly associated with putative target gene, by using find individual motif occurrences (FIMO) with a p-value < 1e-4 to scan a +/− 100bp region around each probe using position weight matrices (PWMs) of human TF motif database JASPAR-Core [85] and Factorbook [86]. Finally, *ELMER* determined the list of upstream regulatory TFs whose expression is associated with TF binding motif DNA methylation. For each motif, we calculated the average DNA methylation of all distal enhancer probes within +/− 100bp of a motif occurrence regions, and correlated with the expression of 1,982 known human TFs [50]. Then we made two groups of samples: M group (20% sample with the highest average DNA methylation for motif) and U group (20% samples with lowest average DNA methylation for motif), for each motif-gene pair. Mann-Whitney *U* test was used to test the null hypothesis that overall gene expression in group *M* was greater or equal than that in group *U*, for each candidate motif-TF pair. This resulted in a raw *p*-value (*P*) for each of the 1,982 TFs, for each motif tested. All TFs were ranked by the -log10(*P*), and those falling within the top 5 % of this ranking were considered candidate upstream regulators.

### Data analysis

All analyses were performed using the R version 3.2.1 [87]. Enrichment analysis was performed using WEB-based Gene SeT AnaLysis Toolkit (WebGestalt) and Ingenuity Pathway Analysis (Ingenuity Systems, Redwood, California, USA).

## SUPPLEMENTARY MATERIALS FIGURES AND DATAS











































## Abbreviations

PC: Pancreatic cancer
TCGA: The Cancer Genome Atlas
ICGC: International Cancer Genome Consortium
PC: pancreatic cancer
dm-CpGs: differentially methylated CpGs
DEG: differentially expressed genes
RSEM: RNAseq by Expectation Maximization
IPA: Ingenuity Pathway Analysis
WebGestalt: WEB-based Gene SeT AnaLysis Toolkit
ELMER: Enhancer Linking by Methylation/Expression Relationship
CpG: Cytosine Guanine dinucleotide
UTR: Untranslated Region
TSS: Transcription Start Site
Mb: Mega base pairs
