# Supplementary material for: Genome-wide DNA methylation analysis reveals molecular subtypes of pancreatic cancer
Source: Oncotarget. 2017 Mar 7;8(17):28990–9012. doi: 10.18632/oncotarget.15993 (PMC5438707; doi:10.18632/oncotarget.15993)
Supplement: Supplementary file 1 [file oncotarget-08-28990-s001.pdf]

Genome-wide DNA methylation analysis reveals molecular subtypes of pancreatic cancer

SUPPLEMENTARY FIGURES AND TABLES

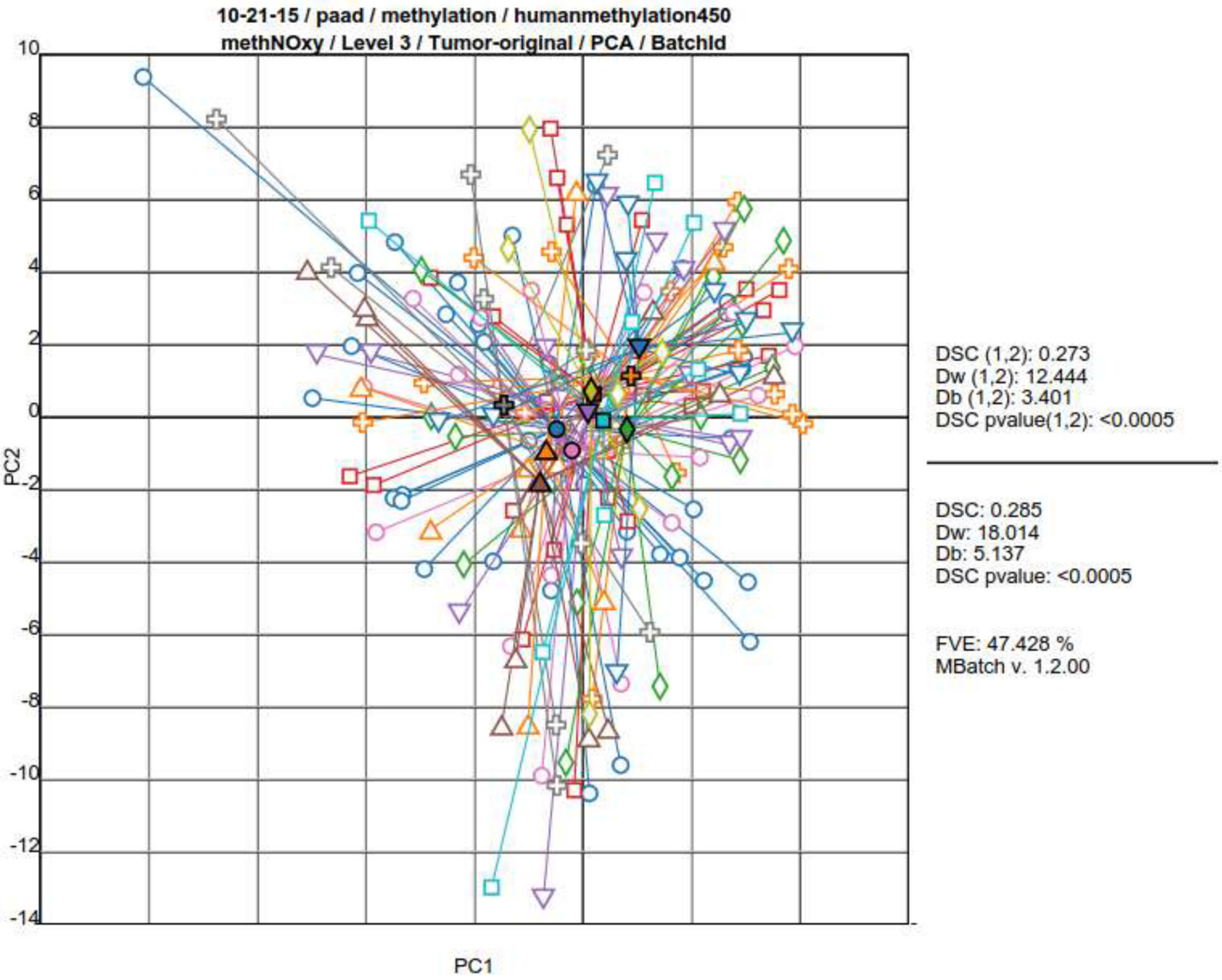

Supplementary Figure 1: PCA plot of TCGA level3 DNA methylation data. Plot suggests that there is no batch effect in level3 TCGA PC HumanMethylation450k BeadChip DNA methylation data.

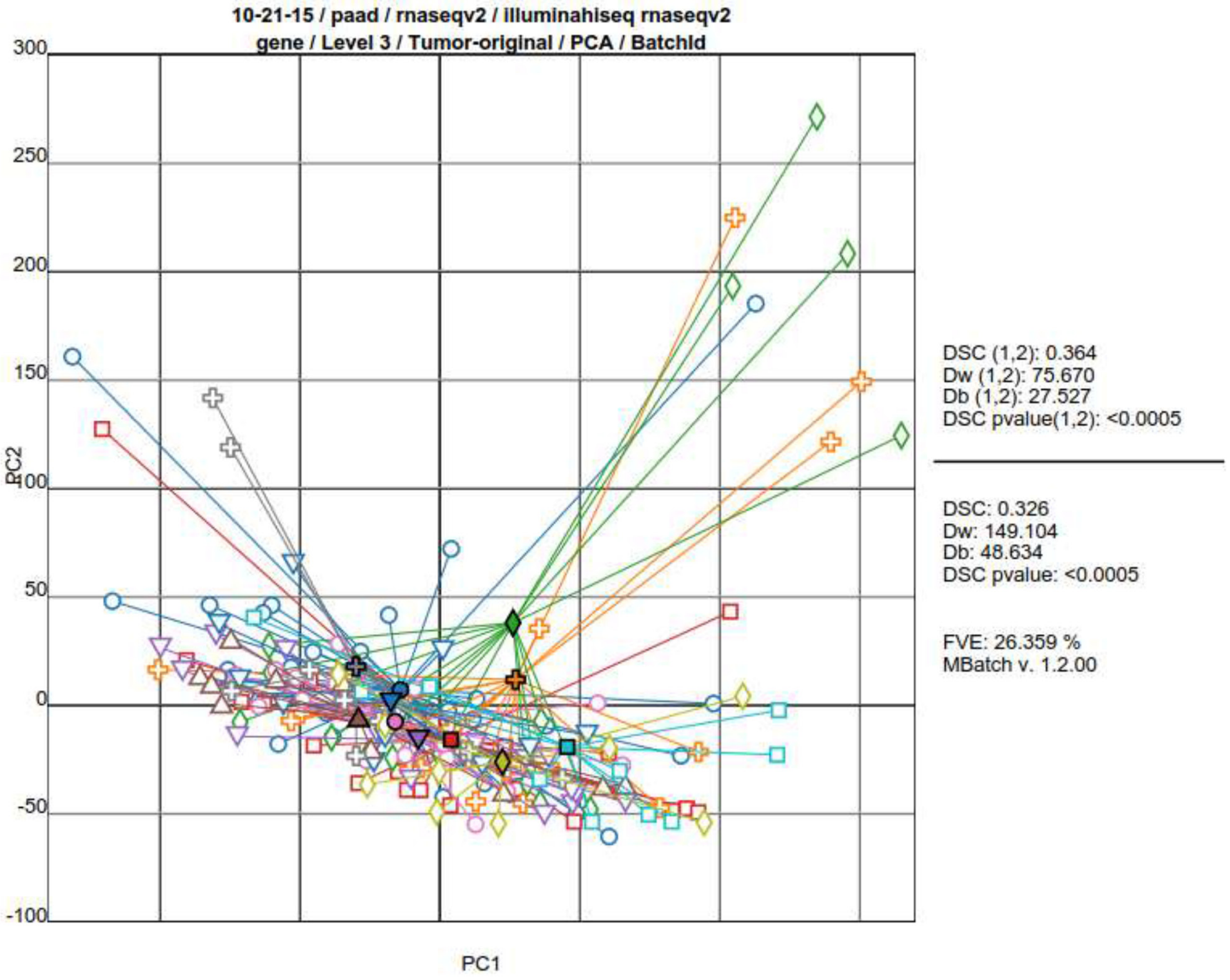

Supplementary Figure 2: PCA plot of TCGA level3 gene-level expression data. This plot shows that there is no batch effect in level3 RNASeqV2 gene expression data.

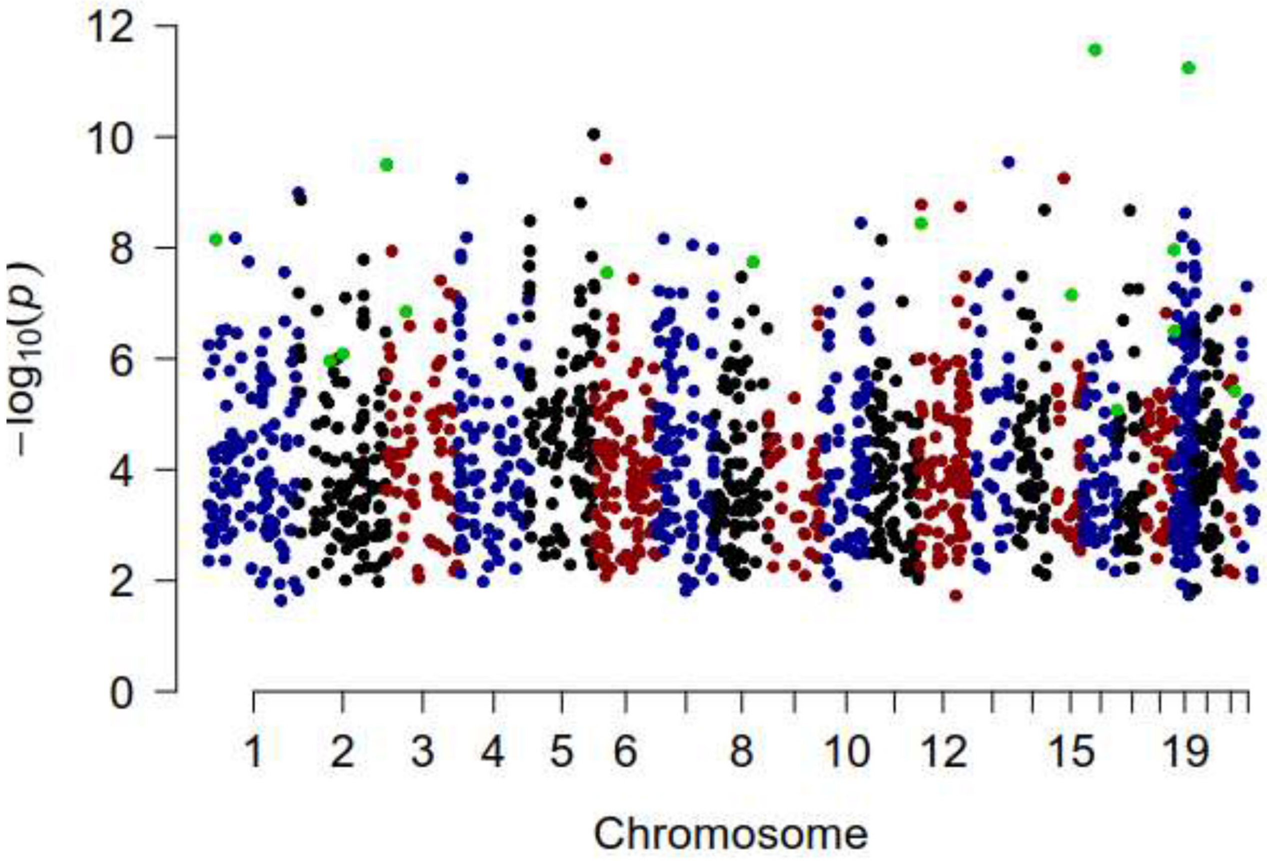

Supplementary Figure 3: Manhattan plot of differentially methylated CpG islands. Hypomethylated CpG island are in green color.

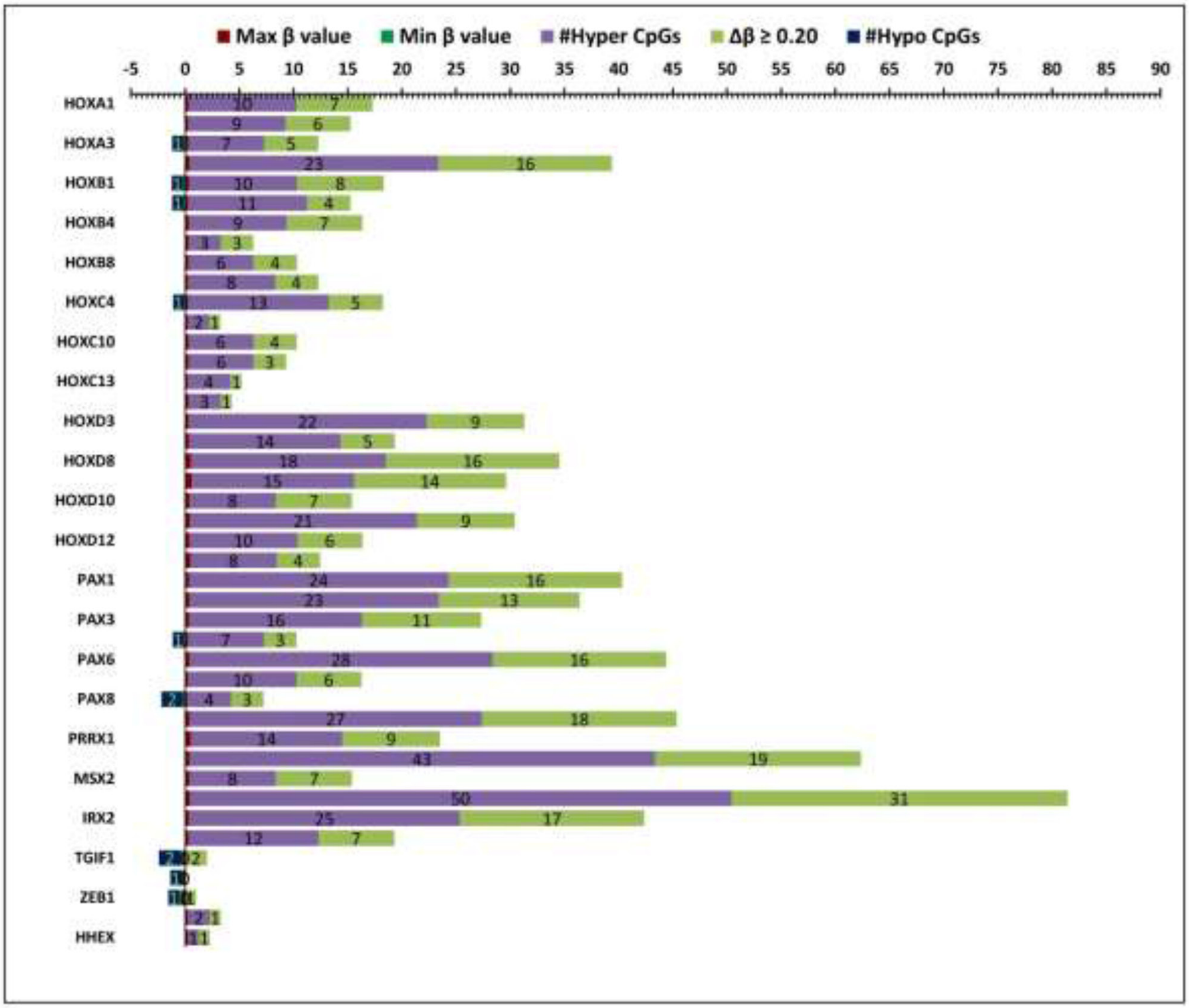

Supplementary Figure 4: DNA methylation of CpG sites in different homeobox-containing gene families in PC. For each homeobox-containing gene we calculated the number of differentially hypermethylated and hypomethylated CpG sites which meet the FDR threshold. Hypermethylated CpGs are on the right side and hypomethylated on the left. Purple color shows the total number of differentially methylated CpG sites, and green color shows the number of CpGs with $\Delta\beta \geq 0.2$.

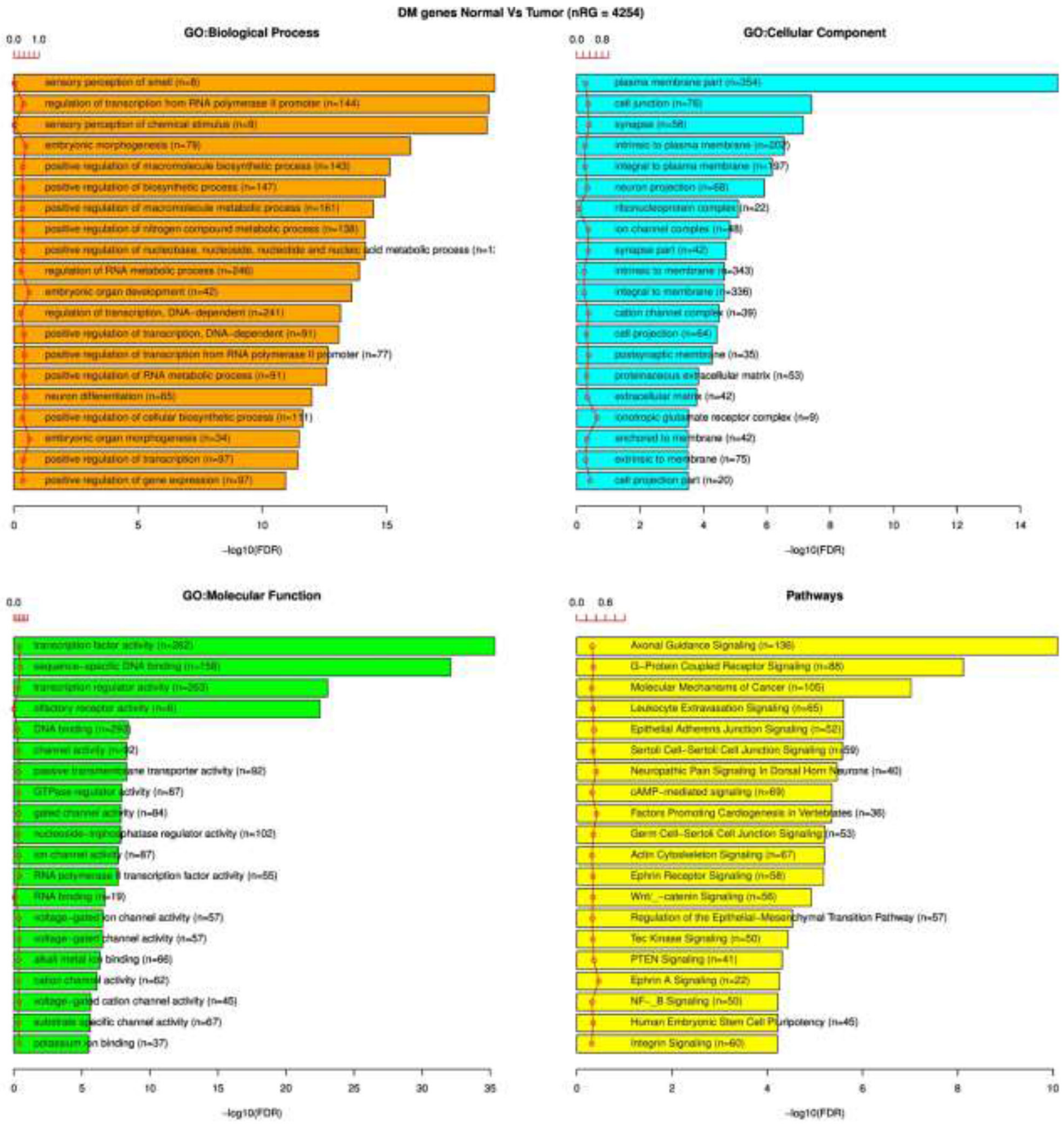

Supplementary Figure 5: GO and pathway enrichment of differentially methylated genes in PC using DAVID in R. We used probes which $\Delta\beta \geq 0.2$, total 4254 genes.

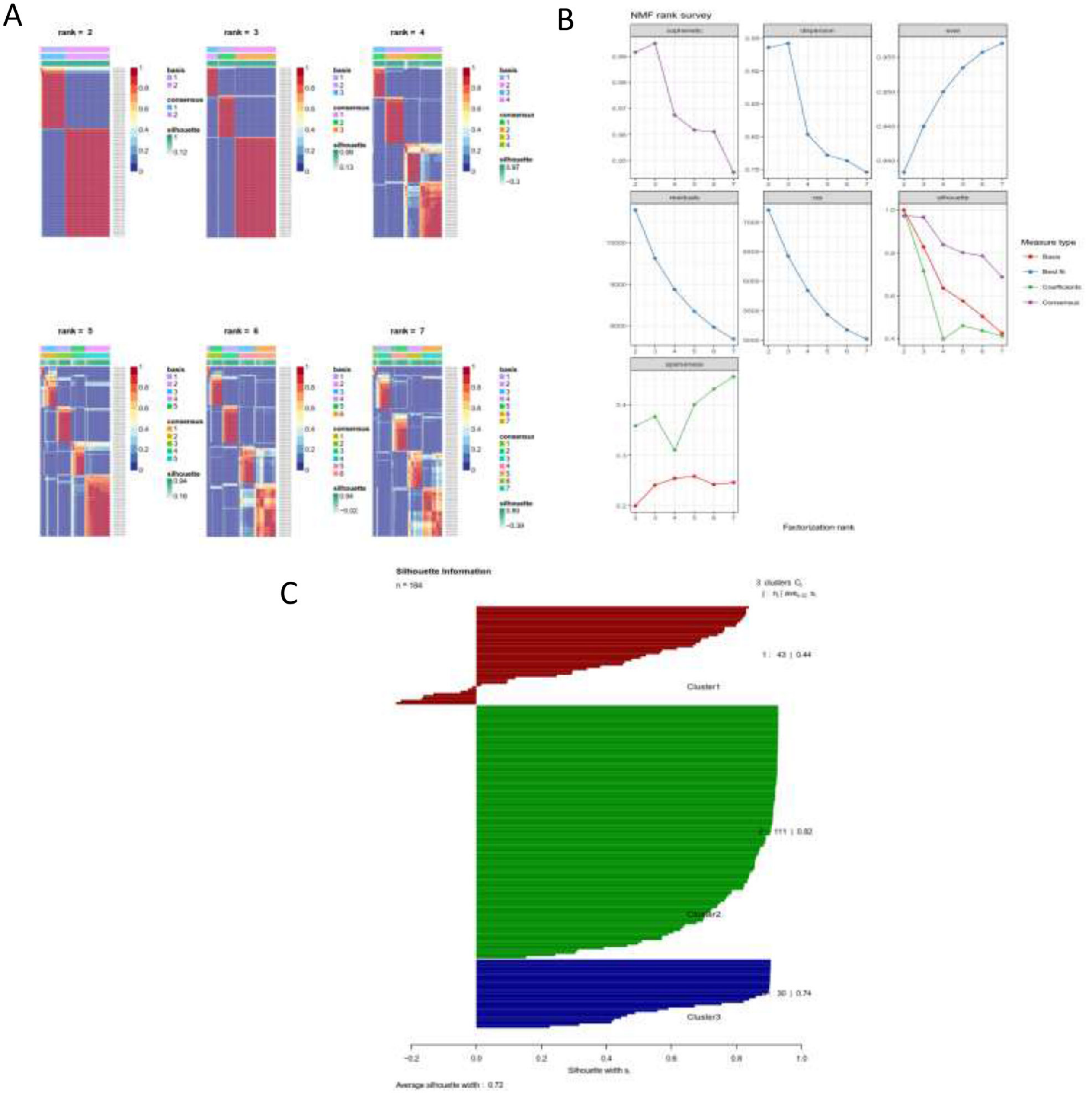

Supplementary Figure 6: NMF clustering result for k 2 to 7. (A) Consensus clustering for different k size after 500 iterations. **(B)** NMF rank survey for k 2 to 7. **(C)** Silhouette score for best k i.e. 3.

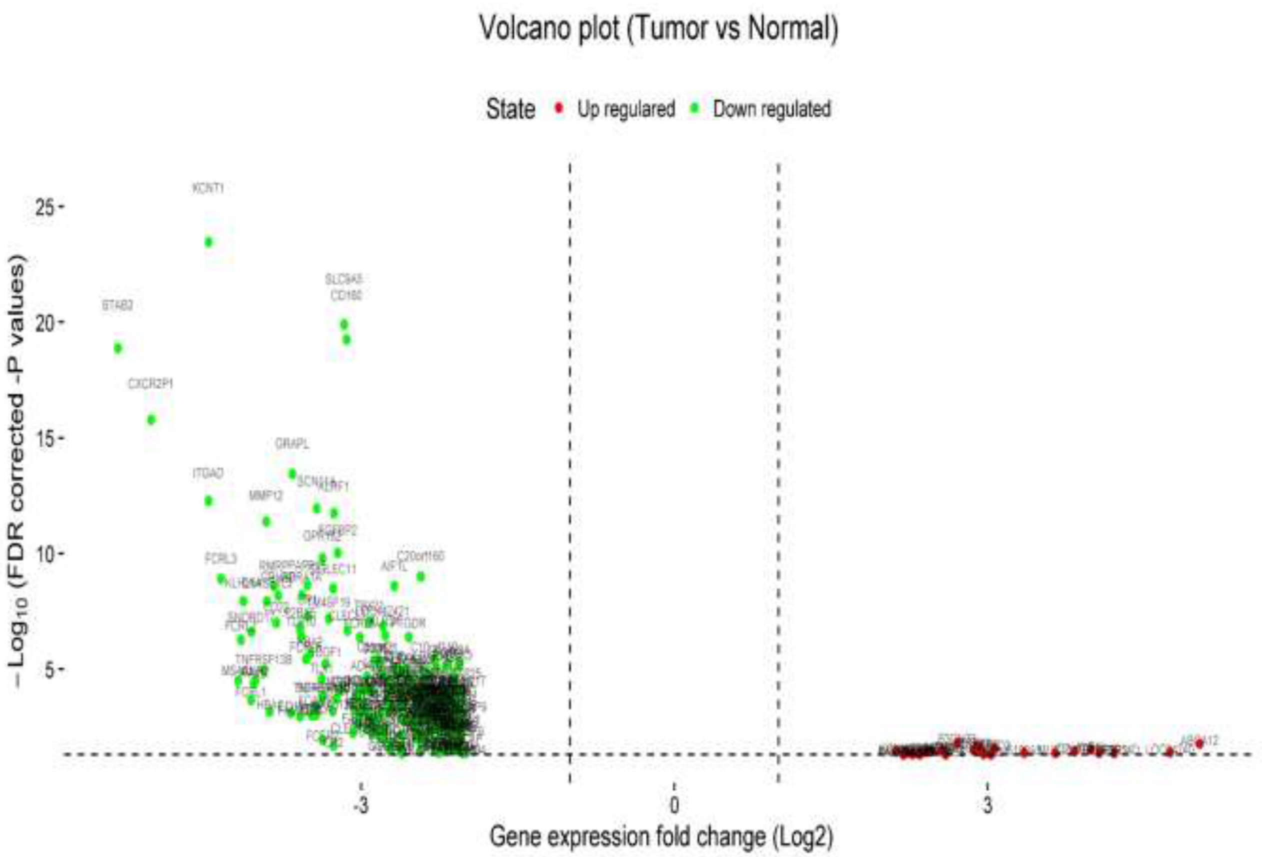

Supplementary Figure 7: Volcano plot of DEG in PC by using edgeR. In this figure we plot differentially methylated genes in PC with FDR 0.05, genes are in red are upregulated and blue are downregulated.

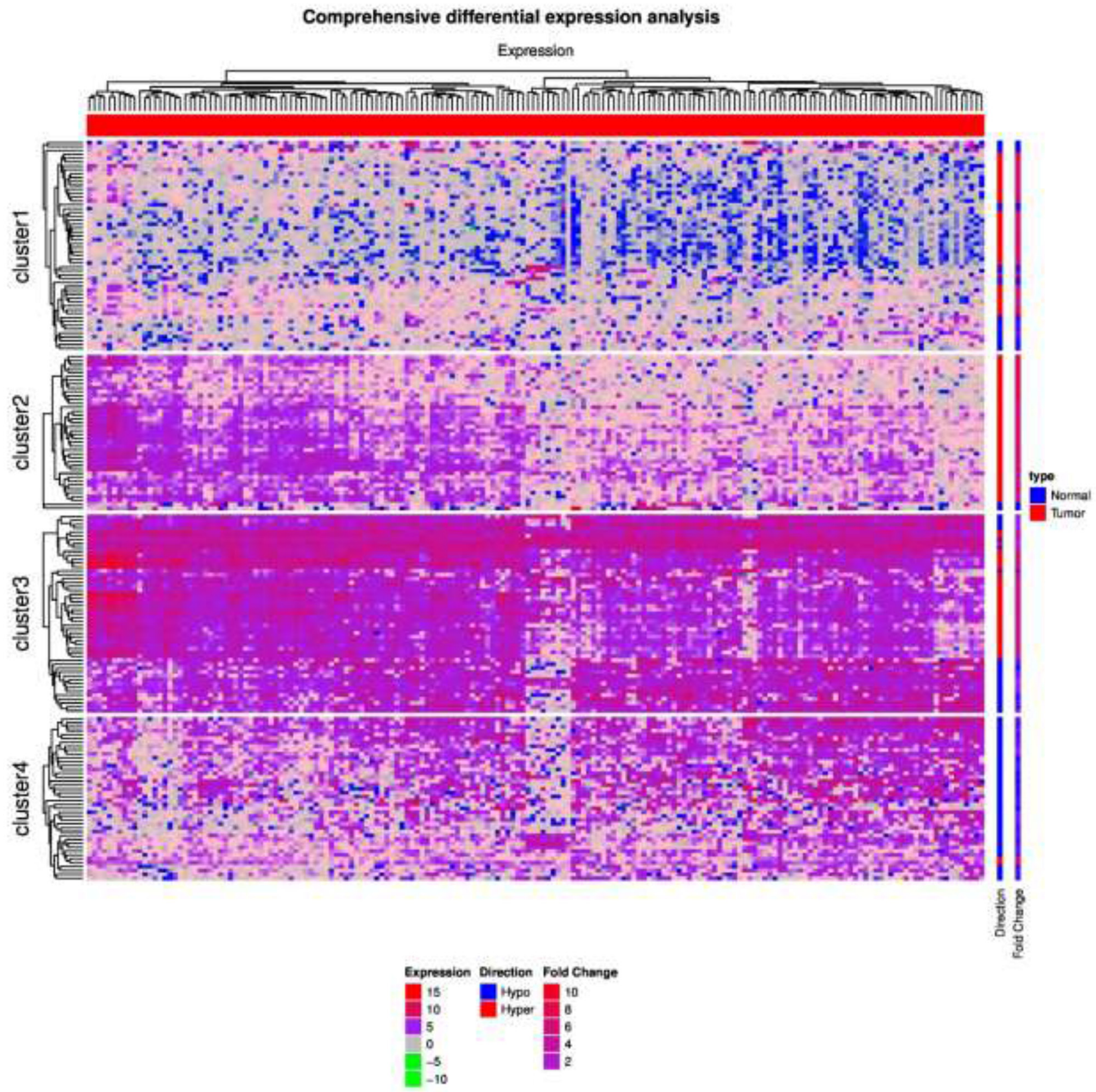

Supplementary Figure 8: Unsupervised clustering of TCGA PC patient's data on the basis of DEG. Figure also has information of direction and fold change in tumor of DEG in TCGA PC.

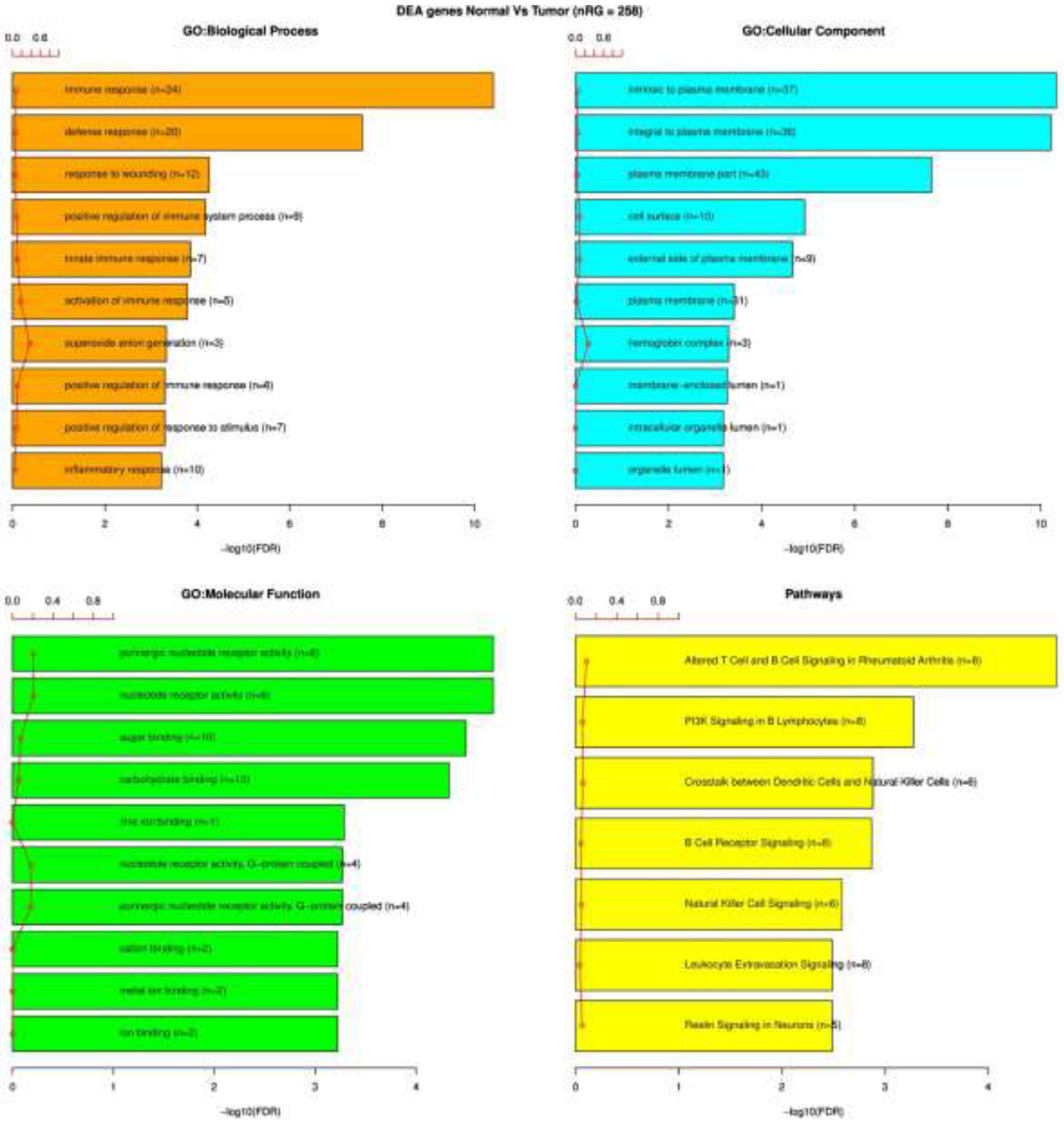

Supplementary Figure 9: GO and pathway enrichment of DEG in PC by using DAVID in R. We used 258 DEG with p-value and adjusted p-value 0.05.

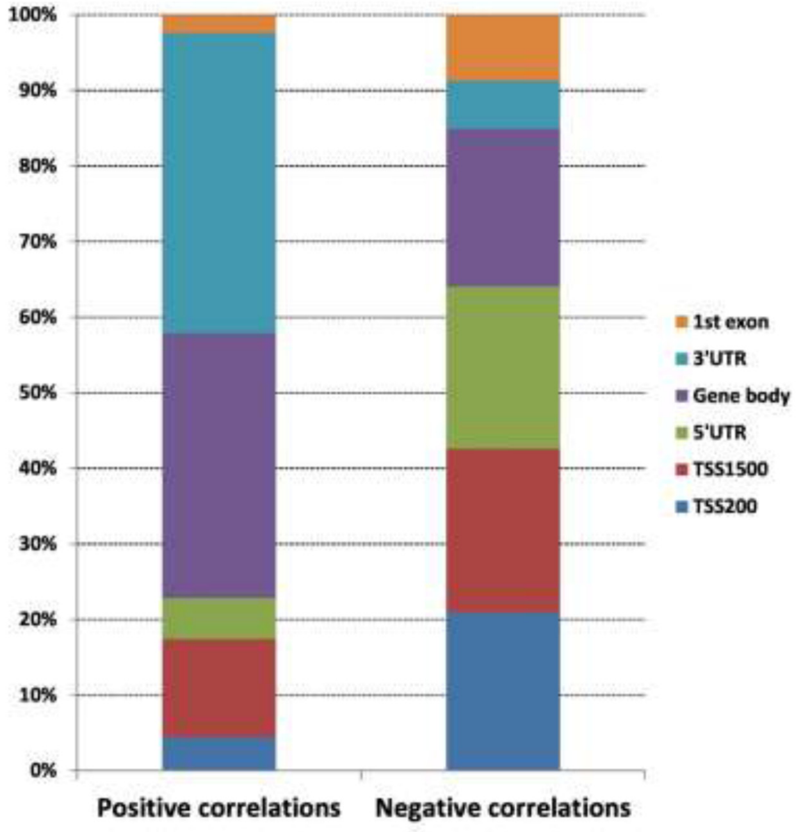

Supplementary Figure 10: Positive and negative correlations of DNA methylation and gene expression. We used Bonferroni corrected P value < 0.05 for stacked plot.

Supplementary Data 1: An excel file listing differentially methylated CpG IDs and its details.

Supplementary File 1

Supplementary Data 2: An excel file listing methylation frequency rate for all chromosomes. This table also has total number of hypermethylated and hypomethylated probes for each chromosomes and hypermethylation and hypomethylation frequency.

Supplementary File 2

Supplementary Data 3: An excel file for total number of hypermethylated and hypomethylated probes for each subregions. This table also have hypermethylation and hypomethylation ratio for each subregions.

Supplementary File 3

Supplementary Data 4: An excel file listing of differentially methylated CpG islands.

Supplementary File 4

Supplementary Data 5: An excel file listing differentially methylated CpGs of epigenetic enzymes, histone proteins and chromatin regulators.

Supplementary File 5

Supplementary Data 6: An excel file listing Homeobox-containing genes dm-CpGs and its details.

Supplementary File 6

Supplementary Data 7: An excel file listing differentially methylated CpGs for pancreatic development and pancreatic signaling related genes.

Supplementary File 7

Supplementary Data 8: An excel file listing KEEG pathways for differentially methylated genes by using WebGestalt.

Supplementary File 8

Supplementary Data 9: An excel file listing of IPA canonical pathways for differentially methylated genes.

Supplementary File 9

Supplementary Data 10: An excel file of enriched GO for differentially methylated genes by using WebGestalt.

Supplementary File 10

Supplementary Data 11: An excel file for listing differentially expressed genes in PC by using FDR 0.05 in edgeR.

Supplementary File 11

Supplementary Data 12: An excel file of listing enriched KEGG pathways for DEG by using WebGestalt.

Supplementary File 12

Supplementary Data 13: An excel file listing IPA canonical pathways for DEGs.

Supplementary File 13

Supplementary Data 14: An excel file of listing WebGestalt based enriched GO for DEGs.

Supplementary File 14

Supplementary Data 15: An excel file for listing probes which have statistically significant correlation with gene expression by using eMap tool.

Supplementary File 15

Supplementary Data 16: An excel file for listing probes which have statistically significant correlation with gene expression by using cor.test in R.

Supplementary File 16

Supplementary Data 17: An excel file for listing homeobox and epigenetic enzyme probes which have statistically significant correlation with gene expression by using cor.test in R.

Supplementary File 17

Supplementary Data 18: An excel file for listing differentially hypomethylated and hypomethylated distal enhancer probes by using ELMER.

Supplementary File 18

Supplementary Data 19: An excel file for listing upstream regulatory transcription factor for hypermethylated and hypomethylated probes.

Supplementary File 19

Supplementary Data 20: An excel file for listing Cytoband of differentially methylated and DEGs.

Supplementary File 20
